# High-resolution melting (HRM)-based detection of polymorphisms in the malic enzyme and glucose-6-phosphate isomerase genes for *Leishmania infantum* genotyping

**DOI:** 10.1186/s13071-023-05878-y

**Published:** 2023-08-14

**Authors:** Gloria Buffi, Marcello Ceccarelli, Aurora Diotallevi, Michelalberto Abruzzese, Federica Bruno, Germano Castelli, Fabrizio Vitale, Francesca Andreoni, Daniela Bencardino, Mauro Magnani, Luca Galluzzi

**Affiliations:** 1 Department of Biomolecular Sciences, University of Urbino Carlo Bo, Urbino, PU Italy; 2 OIE Leishmania Reference Laboratory, Centro di Referenza Nazionale per le Leishmaniosi (C.Re.Na.L.), Istituto Zooprofilattico Sperimentale Della Sicilia, Palermo, PA Italy

**Keywords:** *Leishmania infantum*, Leishmaniasis, MLST, NGS, qPCR, HRM analysis, Malic enzyme, Glucose-6-phosphate isomerase, Genotype

## Abstract

**Background:**

Leishmaniasis is a zoonotic disease endemic in the Mediterranean region where *Leishmania infantum* is the causative agent of human and canine infection. Characterization of this parasite at the subspecies level can be useful in epidemiological studies, to evaluate the clinical course of the disease (e.g. resistant strains, visceral and cutaneous forms of leishmaniasis) as well as to identify infection reservoirs. Multilocus enzyme electrophoresis (MLEE), a method currently recognized as the reference method for characterizing and identifying strains of *Leishmania*, is cumbersome and time-consuming and requires cultured parasites. These disadvantages have led to the development of other methods, such as multilocus microsatellite typing (MLMT) and multilocus sequence typing (MLST), for typing *Leishmania* parasites; however, these methods have not yet been applied for routine use. In this study, we first used MLST to identify informative polymorphisms on single-copy genes coding for metabolic enzymes, following which we developed two rapid genotyping assays based on high-resolution melting (HRM) analysis to explore these polymorphisms in *L. infantum* parasites.

**Methods:**

A customized sequencing panel targeting 14 housekeeping genes was designed and MLST analysis was performed on nine *L. infantum* canine and human strains/isolates. Two quantitative real-time PCR-HRM assays were designed to analyze two informative polymorphisms on malic enzyme (ME) and glucose-6-phosphate isomerase (GPI) genes (390T/G and 1831A/G, respectively). The two assays were applied to 73 clinical samples/isolates from central/southern Italy and Pantelleria island, and the results were confirmed by DNA sequencing in a subset of samples.

**Results:**

The MLST analysis, together with sequences available in the Genbank database, enabled the identification of two informative polymorphisms on the genes coding for ME and GPI. The fast screening of these polymorphisms using two HRM-based assays in 73 clinical samples/isolates resulted in the identification of seven genotypes. Overall, genotype 1 (sequence type 390T/1831G) was the most highly represented (45.2%) in the overall sample and correlated with the most common *L. infantum* zymodemes (MON-1, MON-72). Interestingly, in Pantelleria island, the most prevalent genotype (70.6%) was genotype 6 (sequence type 390T/1831A).

**Conclusions:**

Applying our HRM assays on clinical samples allowed us to identify seven different genotypes without the need for parasite isolation and cultivation. We have demonstrated that these assays could be used as fast, routine and inexpensive tools for epidemiological surveillance of *L. infantum* or for the identification of new infection reservoirs.

**Graphical abstract:**

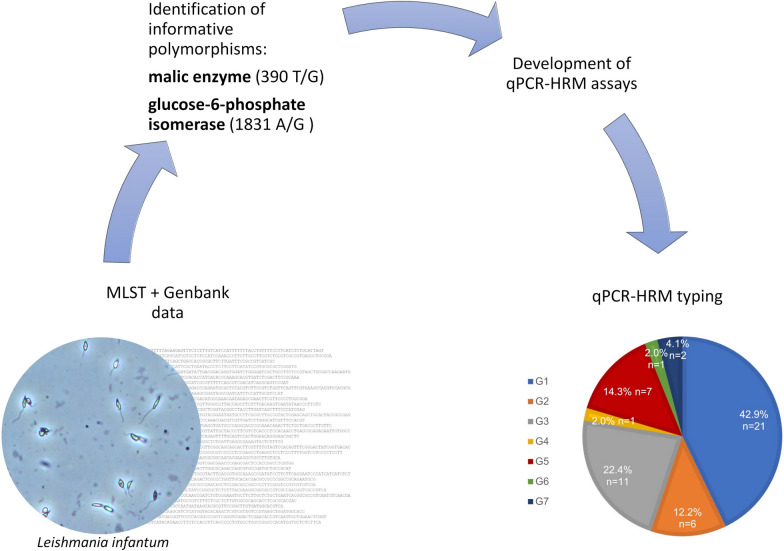

**Supplementary Information:**

The online version contains supplementary material available at 10.1186/s13071-023-05878-y.

## Background

Leishmaniasis is a zoonotic disease endemic in the Mediterranean basin that is mainly caused by *Leishmania infantum,* the etiological agent of human cutaneous and visceral leishmaniasis (CL and VL, respectively), as well as of canine leishmaniasis (CanL). *Leishmania* characterization is traditionally performed by multilocus enzyme electrophoresis (MLEE), which is currently still considered by the WHO to be the reference method for parasite typing [1]. MLEE, which was developed at the Centre for Leishmaniasis of Montpellier (France) (MON system), is based on the different electrophoretic mobilities of 15 metabolic enzymes: malate dehydrogenase (MDH), malic enzyme (ME), isocitrate dehydrogenase (ICD), 6-phosphogluconate dehydrogenase (PGD), glucose-6-phosphate dehydrogenase (G6PD), glutamate dehydrogenase (GLUD), NADH diaphorase (DIA), purine nucleoside phosphorylase 1 (NP1), purine nucleoside phosphorylase 2 (NP2), glutamate–oxaloacetate transaminases 1 and 2 (GOT1, GOT2), phosphoglucomutase (PGM), fumarate hydratase (FH), mannose-phosphate isomerase (MPI) and glucose-6-phosphate isomerase (GPI) [2]. The comparison of isoenzyme mobility with a reference strain has led to the identification of > 300 zymodemes, all referred to using MON terms. Regarding *L. infantum*, 45 zymodemes have been identified. Zoonotic VL and CanL are mainly caused by the MON-1, MON-24, MON-34, MON-72, MON-77, MON-80, MON-98, MON-105, MON-108, MON-199 and MON-199 NP1130 variant and MON-281. In particular, MON-1 is the most frequent zymodeme in humans and dogs [3, 4], followed by MON-72, which is more diffuse in CanL but which has also been identified in human patients [5]. In contrast, some zymodemes (i.e. MON-11, MON-27, MON-28, MON-29, MON-33, MON-189) have been isolated only in humans [6]. Moreover, several non-MON-1 parasites have been reported in patients with HIV [7]. These findings bring into question the role of dogs, historically considered the main reservoir of infection for humans, in the transmission of the disease. In fact, the homogeneity of zymodemes identified in the canine population does not reflect the heterogeneity of those found in humans.

*Leishmania* characterization based on MLEE has a number of limitations: it is time-consuming and cumbersome, requires parasite isolation and cultivation and can be performed only in a few laboratories. To overcome these challenges, various biomolecular approaches have been proposed, including multilocus sequence typing (MLST) [8] and multilocus microsatellite typing (MLMT) [9]. However, despite the robustness of the MLST outcome, the application of this method directly to clinical samples is difficult due to its limited sensitivity: it is based mainly on single-copy genes. Moreover, amplifying several genes in parallel and sequencing them is costly and time-consuming. In comparison, MLMT has been used on clinical samples [9], but the method requires several PCR amplifications and capillary gel electrophoresis analysis. In summary, these two techniques are time-consuming and relatively expensive, and may require parasite isolation; therefore, their applicability in epidemiological studies and clinical routines can be challenging.

 In this context, in a previous study we developed an alternative approach based on a high-resolution melting (HRM) assay to differentiate the most common zymodemes (i.e. MON-1, MON-72, MON-201 [except for MHOM/TN/80/IPT1, a MON-1 zymodeme from Tunisia]) that exploits the polymorphism 390T>G in the malic enzyme (ME) gene, evidencing a partial agreement, although not univocal, between genotyping results and MLEE results [10]. In the present study, which represents an extension of the previous one, we designed an MLST panel on 14 genes encoding enzymes used for MLEE with the aims: (i) to identify further polymorphisms useful for *L. infantum* typing; and (ii) to develop and apply HRM-based tests on the most informative polymorphisms to implement the rapid and cheap characterization of *L. infantum* strains.

## Methods

### Sample collection

*Leishmania infantum* strains, isolates and clinical samples analyzed in this study are listed in Table 1. The clinical samples consisted of peripheral blood (obtained by venipuncture in EDTA tubes); buffy-coat (i.e. white cells collected after whole blood centrifugation at 210 *g *for 10 min); bone marrow and lymph node needle aspirates (harvested in EDTA tubes for blood collection); skin scrapings and skin biopsy on cutaneous lesions (obtained using a sterile cotton swab and a biopsy punch, respectively) collected in sterile tubes containing phosphate-buffered saline (PBS); and conjunctival swabs (i.e. exfoliative epithelial cells collected from the lower conjunctival sac using sterile cotton swabs). Clinical isolates were obtained from fresh lymph node aspirates or skin biopsy, as described previously [11]. Clinical samples and isolates were collected between 2015 and 2021 from central and southern Italy; among them, a fraction came from a canine population resident in a kennel on Pantelleria Island, a small island (80 km^2^) located southwest of Sicily and 60 km east of the Tunisian coast.

**Table 1:** *Leishmania infantum* strains, clinical isolates and clinical samples analyzed in this study

Sample number	Sample ID	Type of sample	Zymodeme	Host	Geographic origin
1	MCAN/ES/98/LLM-724^1^	Reference strain	MON-1	Dog	Spain
2	MHOM/PT/2000/IMT260^1^	Reference strain	MON-1	Human	Portugal
3	MHOM/ES/1986/BCN16^1^	Reference strain	MON-1	Human	Spain
4	MHOM/FR/1997/LSL29^1^	Reference strain	MON-1	Human	France
5	MHOM/ES/1993/PM1^1^	Reference strain	MON-1	Human	Spain
6	MHOM/FR/1995/LPN114^1^	Reference strain	MON-1	Human	France
7	MHOM/FR/1996/LEM3249^1^	Reference strain	MON-29	Human	France
8	MHOM/ES/1991/LEM2298^1^	Reference strain	MON-183	Human	Spain
9	MHOM/ES/1988/LLM175^1^	Reference strain	MON-198	Human	Spain
10	MHOM/IT/1994/ISS1036^1^	Reference strain	MON-228	Human	Italy
11	MHOM/ES/1992/LLM373^1^	Reference strain	MON-199	Human	Spain
12	MHOM/MT/1985/BUCK^1^	Reference strain	MON-78	Human	Malta
13	MHOM/FR/78/LEM75^2^	Reference strain	MON-1	Human	France
14	MHOM/TN/80/IPT1^2^	Reference strain	MON-1	Human	Tunisia
15	MHOM/DZ/82/LIPA59	Reference strain	MON-24	Human	Algeria
16	MHOM/ES/81/BCN1	Reference strain	MON-29	Human	Spain
17	MHOM/IT/86/ISS218	Reference strain	MON-72	Human	Italy
18	MHOM/IT/93/ISS822	Reference strain	MON-201	Human	Italy
19	MHOM/IT/08/31U^2^	Reference strain	MON-1	Human	Palermo (Italy)
20	MHOM/IT/08/49U	Reference strain	MON-1	Human	Palermo (Italy)
21	Pan-30^2^	Clinical isolate (lymph node aspirate)		Dog	Pantelleria (Italy)
22	Pan-42^2^	Clinical isolate (lymph node aspirate)		Dog	Pantelleria (Italy)
23	Pan-64^2^	Clinical isolate (lymph node aspirate)		Dog	Pantelleria (Italy)
24	10816	Clinical isolate (lymph node aspirate)	MON-1	Cat	Palermo (Italy)
25	V2921	Clinical isolate (spleen)	MON-1	Marten	Palermo (Italy)
26	791	Clinical isolate (lymph node aspirate)	MON-1	Cat	Messina (Italy)
27	MHOM/IT/2019/cur-1^2^	Clinical isolate (cutaneous biopsy)		Human	Pesaro-Urbino (Italy)
28	Elr-sci^2^	Clinical isolate (lymph node aspirate)		Dog	Pesaro-Urbino (Italy)
29	Bra-aii^2^	Clinical isolate (lymph node aspirate)		Dog	Pesaro-Urbino (Italy)
30	Plo-roi	Clinical sample (bone marrow)		Dog	Pesaro-Urbino (Italy)
31	Aro-sai	Clinical sample (conjunctival swab)		Dog	Pesaro-Urbino (Italy)
32	Els-mai^a^	Clinical sample (conjunctival swab)		Dog	Pesaro-Urbino (Italy)
33	Els-mai^b^	Clinical sample (conjunctival swab)		Dog	Pesaro-Urbino (Italy)
34	Toy-gai	Clinical sample (lymph node aspirate)		Dog	Pesaro-Urbino (Italy)
35	Koa-cro	Clinical sample (lymph node aspirate)		Dog	Pesaro-Urbino (Italy)
36	Zeo-sci	Clinical sample (lymph node aspirate)		Dog	Pesaro-Urbino (Italy)
37	Gia-spi	Clinical sample (conjunctival swab)		Dog	Pesaro-Urbino (Italy)
38	Grg-rao	Clinical sample (buffy coat)		Dog	Pesaro-Urbino (Italy)
39	Vea-fri	Clinical sample (bone marrow)		Dog	Pesaro-Urbino (Italy)
40	Jon-doe	Clinical sample (peripheral blood)		Dog	Pesaro-Urbino (Italy)
41	Pan-1	Clinical sample (lymph node aspirate)		Dog	Pantelleria (Italy)
42	Pan-2	Clinical sample (lymph node aspirate)		Dog	Pantelleria (Italy)
43	Pan-4	Clinical sample (lymph node aspirate)		Dog	Pantelleria (Italy)
44	Pan-5	Clinical sample (lymph node aspirate)		Dog	Pantelleria (Italy)
45	Pan-6	Clinical sample (lymph node aspirate)		Dog	Pantelleria (Italy)
46	Pan-9	Clinical sample (lymph node aspirate)		Dog	Pantelleria (Italy)
47	Pan-10	Clinical sample (lymph node aspirate)		Dog	Pantelleria (Italy)
48	Pan-11	Clinical sample (lymph node aspirate)		Dog	Pantelleria (Italy)
49	Pan-12	Clinical sample (lymph node aspirate)		Dog	Pantelleria (Italy)
50	Pan-13	Clinical sample (lymph node aspirate)		Dog	Pantelleria (Italy)
51	Pan-14	Clinical sample (lymph node aspirate)		Dog	Pantelleria (Italy)
52	Pan-15	Clinical sample (lymph node aspirate)		Dog	Pantelleria (Italy)
53	Pan-16	Clinical sample (lymph node aspirate)		Dog	Pantelleria (Italy)
54	Pan-21	Clinical sample (lymph node aspirate)		Dog	Pantelleria (Italy)
55	Pan-22	Clinical sample (lymph node aspirate)		Dog	Pantelleria (Italy)
56	Pan-24	Clinical sample (lymph node aspirate)		Dog	Pantelleria (Italy)
57	Pan-25	Clinical sample (lymph node aspirate)		Dog	Pantelleria (Italy)
58	Pan-26	Clinical sample (lymph node aspirate)		Dog	Pantelleria (Italy)
59	Pan-27	Clinical sample (lymph node aspirate)		Dog	Pantelleria (Italy)
60	Pan-28	Clinical sample (lymph node aspirate)		Dog	Pantelleria (Italy)
61	Psalb	Clinical sample (buffy coat)		Human	Pesaro-Urbino (Italy)
62	Dae-dio	Clinical sample (cutaneous biopsy)		Human	Pesaro-Urbino (Italy)
63	Mao-pai	Clinical sample (skin scraping)		Human	Pesaro-Urbino (Italy)
64	Gae-bea	Clinical sample (skin scraping)		Human	Pesaro-Urbino (Italy)
65	Kua-asn	Clinical sample (skin scraping)		Human	Pesaro-Urbino (Italy)
66	1038U	Clinical sample (peripheral blood)		Human	Messina (Italy)
67	1522U	Clinical sample (peripheral blood)		Human	Catania (Italy)
68	1536U	Clinical sample (peripheral blood)		Human	Messina (Italy)
69	1538U	Clinical sample (peripheral blood)		Human	Messina (Italy)
70	1578U	Clinical sample (peripheral blood)		Human	Catania (Italy)
71	1758U	Clinical sample (skin scraping)		Human	Palermo (Italy)
72	1759U	Clinical sample (skin scraping)		Human	Agrigento (Italy)
73	1761U	Clinical sample (skin scraping)		Human	Agrigento (Italy)
74	1810U	Clinical sample (peripheral blood)		Human	Agrigento (Italy)
75	2000U	Clinical sample (skin scraping)		Human	Agrigento (Italy)
76	2068U	Clinical sample (skin scraping)		Human	Palermo (Italy)
77	2073U	Clinical sample (skin scraping)		Human	Agrigento (Italy)
78	2579U	Clinical sample (peripheral blood)		Human	Palermo (Italy)
79	2596U	Clinical sample (peripheral blood)		Human	Palermo (Italy)
80	2602U	Clinical sample (peripheral blood)		Human	Ragusa (Italy)
81	2604U	Clinical sample (peripheral blood)		Human	Agrigento (Italy)
82	2618U	Clinical sample (peripheral blood)		Human	Messina (Italy)
83	2619U	Clinical sample (peripheral blood)		Human	Messina (Italy)
84	2626U	Clinical sample (peripheral blood)		Human	Agrigento (Italy)
85	2629U	Clinical sample (peripheral blood)		Human	Agrigento (Italy)
86	2632U	Clinical sample (cutaneous biopsy)		Human	Messina (Italy)
87	2647U	Clinical sample (cutaneous biopsy)		Human	Messina (Italy)
88	2652U	Clinical sample (peripheral blood)		Human	Catania (Italy)
89	2660U	Clinical sample (skin scraping)		Human	Messina (Italy)
90	2668U	Clinical sample (peripheral blood)		Human	Enna (Italy)
91	2669U	Clinical sample (peripheral blood)		Human	Catania (Italy)
92	2746U	Clinical sample (skin scraping)		Human	Palermo (Italy)
93	2897U	Clinical sample (skin scraping)		Human	Palermo (Italy)

*MLST* Multilocus sequence typing, *MON* parasite typing system of the Centre for Leishmaniasis of Montpellier (France)

^1^Sample sequences available on the database and published in Ceccarelli et al. [10]

^2^Selected for MLST analysis

Els-mai^a^ was a left conjunctival swab; Els-mai^b^ was a right conjunctival swab

Sample numbers 13–26, 41–60 and 66–93 (Table 1) were provided by the OIE Reference Laboratory National Reference Centre for Leishmaniasis (C.Re.Na.L.) (Palermo, Italy) [12, 13]. Additional human samples (nos. 27, 61–65; Table 1) were obtained from the Unit of Infectious Diseases, Marche Nord Hospital (Pesaro, Italy). The parasite isolation of sample 27 (Table 1) was performed as previously described [14, 15]. The canine clinical samples (nos. 28–40; Table 1) were provided from the veterinary clinic “Santa Teresa” in Fano (Marche region, Italy). All samples were collected as part of the diagnostic procedure during routine examination, with the exception of the conjunctival swabs, which were collected for research purposes (see Ethics approval and consent to participate declaration at the end of the article).

All human and veterinary samples were diagnosed as positive for *Leishmania* spp. through objective evaluation, serological tests [i.e. IFAT, SNAP tests (IDEXX Laboratories, Westbrook, ME, USA) or the Speed Leish K test (BVT Groupe Virbac, La Seyne sur Mer, France)] and/or by cytohistological examination. Moreover, after extraction of DNA (see following section), sample positivity was evaluated with qPCR–ML analysis [16]. To confirm the presence of *L. infantum* species, molecular identification was performed with qPCR-based test [17] or internal transcriber spacer 1-PCR restriction fragment length polymorphism (ITS1–PCR RFLP) according to Schönian et al. [18]. Briefly, the PCR products were digested with 10 U HaeIII (Thermo Fisher Scientific, Waltham, MA, USA) at 37 °C for 3 h. The restriction fragments were visualized on 3.5% high-resolution MetaPhor (Cambrex Corp., East Rutherford, NJ, USA) agarose gel.

### DNA extraction

DNA from cultured parasites (reference strains or clinical isolates) and clinical samples (with the exception of conjunctival swabs) were extracted using the DNeasy Blood & Tissue kit (Qiagen, Hilden, Germany) following the manufacturer’s protocol, and subsequently quantified using a Qubit fluorometer (Life Technologies, Thermo Fisher Scientific). DNA from conjunctival swabs was obtained from raw lysates as described previously [19]. Briefly, swabs were incubated for 2 h at 56 °C in 200 μl of lysis buffer (10 mM Tris–HCl pH 8.3, 50 mM KCl, 0.5% Nonidet P40, 0.5% Tween 20, 0.1 mg/ml proteinase K). After swab elimination, the samples were incubated for 10 min at 95 °C and centrifuged at 10,000 *g* for 10 min.

### MLST panel design, library preparation and next-generation sequencing

A total of 14 protein-coding genes were selected for the MLST assay: elongation initiation factor 2alpha, spermidine synthase 1, ICD, GPI, inosine-guanine nucleoside hydrolase, nonspecific nucleoside hydrolase, ME, phosphomannose isomerase, G6PD, malate dehydrogenase, arginase, 6-phosphogluconate dehydrogenase, phosphomannomutase and UDP-N-acetylglucosamine-dolichyl-phosphate N-acetylglucosaminephosphotransferase. A customized sequencing panel was designed with Ion AmpliSeq™ designer v7.0.6 tool (Thermo Fisher Scientific), using the *L. infantum* JPCM5 genome (assembly GCA_900180445.1) as reference (Additional file 1: Table S1). This panel consisted of two primer pools with 88 different amplicons. The nine *Leishmania* strains/isolates sequenced are shown in Table 1 (superscript '2': 5 canine isolates from Pantelleria Island and central Italy, and 4 human isolates/strains from Tunisia, France, central and southern Italy). The DNA library preparation was performed by Ion AmpliSeq™ Library Kit Plus (Thermo Fisher Scientific), starting from 5 ng of DNA. The sequencing of libraries was performed using the Ion Torrent S5 instrument (Thermo Fisher Scientific). After sequencing, reads were mapped to *L. infantum* JPCM5 genome (release version TriTrypDB-45_LinfantumJPCM5_Genome) using the Torrent Browser. Variants were called using LoFreq in Galaxy (Galaxy Version 2.1.5 + galaxy0) [20] using default settings. Integrative Genomics Viewer or Ugene were used for variant visualization. Subsequently, a molecular phylogenetic analysis by the maximum likelihood method based on the General Time Reversible model [21] was performed using MEGAX software.

Polymorphism Information Content (PIC) for single nucleotide polymorphisms (SNPs) were calculated as for dominant markers using the simplified equation described in Serrote et al. [22].

### Primer design

PCR products encompassing informative polymorphisms on the ME and GPI genes (i.e. 390T/G and 1831A/G, as in reference sequences DQ449701.1 and AJ620617.1, respectively) were obtained using a nested approach with four primer pairs (Table 2). The two external primer pairs (including a previously published primer pair [10]) were used to perform the pre-amplification step with conventional PCR (cPCR), while the two internal primer pairs were used for the second amplification step with qPCR, followed by HRM analysis. Primers were designed with Primer-BLAST [23, 24] using *L. infantum* MHOM/FR/78/LEM75 malic enzyme and MHOM/TN/80/IPT1 GPI sequences as reference. The primer positions on the target genes and the polymorphic nucleotides are shown in Additional file 2: Figure S1 and Additional file 3: Figure S2.

**Table 2:** Primers used in this study

Target gene	Reference sequence	Primer	Sequence	PCR assay
Malic enzyme	DQ449701.1	MEint-F	TCAGAACCTTCGCAAGACGA	cPCR-MEint [10]
		MEint-R	CACTTGCCGATGCTGATGC	
		MEint-F	TCAGAACCTTCGCAAGACGA	qPCR-ME65
		ME65-R	GGCCGAGAATGCGGGAG	
Glucose-6-phosphate isomerase	AJ620617.1	GPIext-F	CTCAAGTCCGGCAACATCGT	cPCR-GPIext
		GPIext-R	ACATGCACTTCGCAGCTCT	
		GPI88-F	ACGAACGGCCTGATCAACAT	qPCR-GPI88
		GPI88-R	ACATGCACTTCGCAGCTCTA	

*cPCR* conventional PCR,* F* forward, * GPI* glucose-6-phosphate isomerase, *ME* malic enzyme, * qPCR* quantitative real-time PCR,* R* reverse

### Pre-amplification step by cPCR

To ensure the amplification of clinical samples with low parasite load, we included a pre-amplification step in the cPCR with external primers. The cPCR was performed in duplicate in a final reaction volume of 25 μl containing 2 μl of template DNA, 200 μM dNTP, 2.5 mM MgCl_2_, 200 nM of each primer and 1 U of Hot-Rescue DNA polymerase (Diatheva s.r.l., Fano, Italy). Amplification was carried out in a GeneAmp® PCR System 2700 thermocycler (Applied Biosystems, Thermo Fisher Scientific), with a thermal cycling profile of 94 °C for 7 min, followed by 15 cycles at 94 °C for 30 s, 60 °C for 20 s and 72 °C for 15 s. A no-template tube was included as a negative control.

### qPCR and HRM analyses

The qPCR-ME65 and qPCR-GPI88 assays resulted in amplified products of 65 and 88 bp, respectively, encompassing the polymorphisms 390T/G (qPCR-ME65) and 1831A/G (qPCR-GPI88), respectively. All qPCRs were carried out in a reaction volume of 25 μl containing 1 μl of template DNA (purified genomic DNA for control samples and pre-amplified PCR product for clinical samples) and 12.5 μl of TB Green PreMix ex Taq II Master Mix (Takara Bio Europe, Saint-Germain-en-Laye, France), with 200 nM of each primer. The qPCR reactions were performed in a Rotor-Gene 6000 instrument (Corbett Life Science, Mortlake, Australia), with a thermal cycling profile of 94 °C for 10 min, followed by 45 cycles at 94 °C for 20 s, 60 °C for 20 s and 72 °C for 20 s. Each sample was run in duplicate, and no template control was processed in each run. After amplification, HRM analysis was performed over the range 77–88 °C, at increases of 0.1 °C/s and waiting for 2 s at each temperature. Raw HRM curves were normalized by the Rotor-gene 6000 v.1.7 software (Corbett Life Science). In the qPCR-ME65 assay, MHOM/FR/78/LEM75 and MHOM/TN/80/IPT1 strains were used as references for genotypes 390T and 390G, respectively; in qPCR-GPI88 assay, MHOM/FR/78/LEM75, MHOM/TN/80/IPT1 and MHOM/IT/93/ISS822 strains were used as reference for the genotypes 1831G, 1831A and 1831R (heterozygote), respectively. The assignment of the 390T/G and 1831A/G genotypes was performed by the instrument software with a confidence ≥ 85%.

### Specificity and sensitivity of the qPCR-ME65 and qPCR-GPI88

To evaluate the specificity of the new primer pairs (MEint-F/ME65-R, GPIext-F/GPIext-R and GPI88-F/GPI88-R), we performed qPCR as described above using 1 × 10^–2^ ng of *L. infantum* DNA (strains MHOM/FR/78/LEM75 and MHOM/TN/80/IPT1) and 30 ng of human, canine, feline and *Trypanosoma cruzi* DNA as template. Amplified fragments were analyzed by 1.8% agarose gel electrophoresis with Midori Green Advance DNA stain (NIPPON Genetics EUROPE GmbH, Düren, Germany). The gels were visualized under UV light using a gel doc apparatus (Bio-Rad Laboratories, Hercules, CA, USA). The GeneRuler 100 bp Plus DNA Ladder (Thermo Fisher Scientific) was included as size standard.

To estimate the sensitivity and applicability of the ME65 and GPI88 qPCRs on clinical samples, we established standard curves using serial dilutions of MHOM/FR/78/LEM75 DNA, ranging from 1 × 10^0^ to 1 × 10^−5^ ng per reaction tube. To evaluate the potential interference of host DNA as background in the qPCR assay, each qPCR reaction tube was spiked with 30 ng of human and canine DNA purified from human and canine cell lines (MCF7 and DH82, respectively). The standard curves were obtained from two independent experiments performed in duplicate.

### PCR product sequencing

To confirm the genotype assigned by the Rotorgene software, the amplification products obtained with external primers were purified using the MinElute PCR Purification Kit (Qiagen, Hilden, Germany) and directly sequenced. Overall, 23 and 44 PCR products were sequenced for the ME and GPI genes, respectively. DNA sequencing was performed using the BigDye Terminator v.11 Cycle Sequencing Kit on an ABI PRISM 310 Genetic Analyzer (both Applied Biosystems, Thermo Fisher Scientific). The electropherograms were analyzed using the BioEdit Sequence Alignment Editor [25]. The heterozygous genotype was attributed when two different overlapping peaks were observed in the same position.

### Statistical analyses

To evaluate the different HRM temperatures among the genotypes, we performed a Kruskal–Wallis test, followed by Dunn's multiple comparisons test. All statistical tests were performed using GraphPad Prism version 8.0.2 (GraphPad Software, Inc., La Jolla, CA, USA). A* p*-value ≤ 0.05 was considered to indicate statistical significance. The results are shown as the average of technical replicates for each sample from at least two independent experiments ± standard deviation (SD).

## Results

### MLST on *L. infantum* strains and isolates

**Fig. 1:**
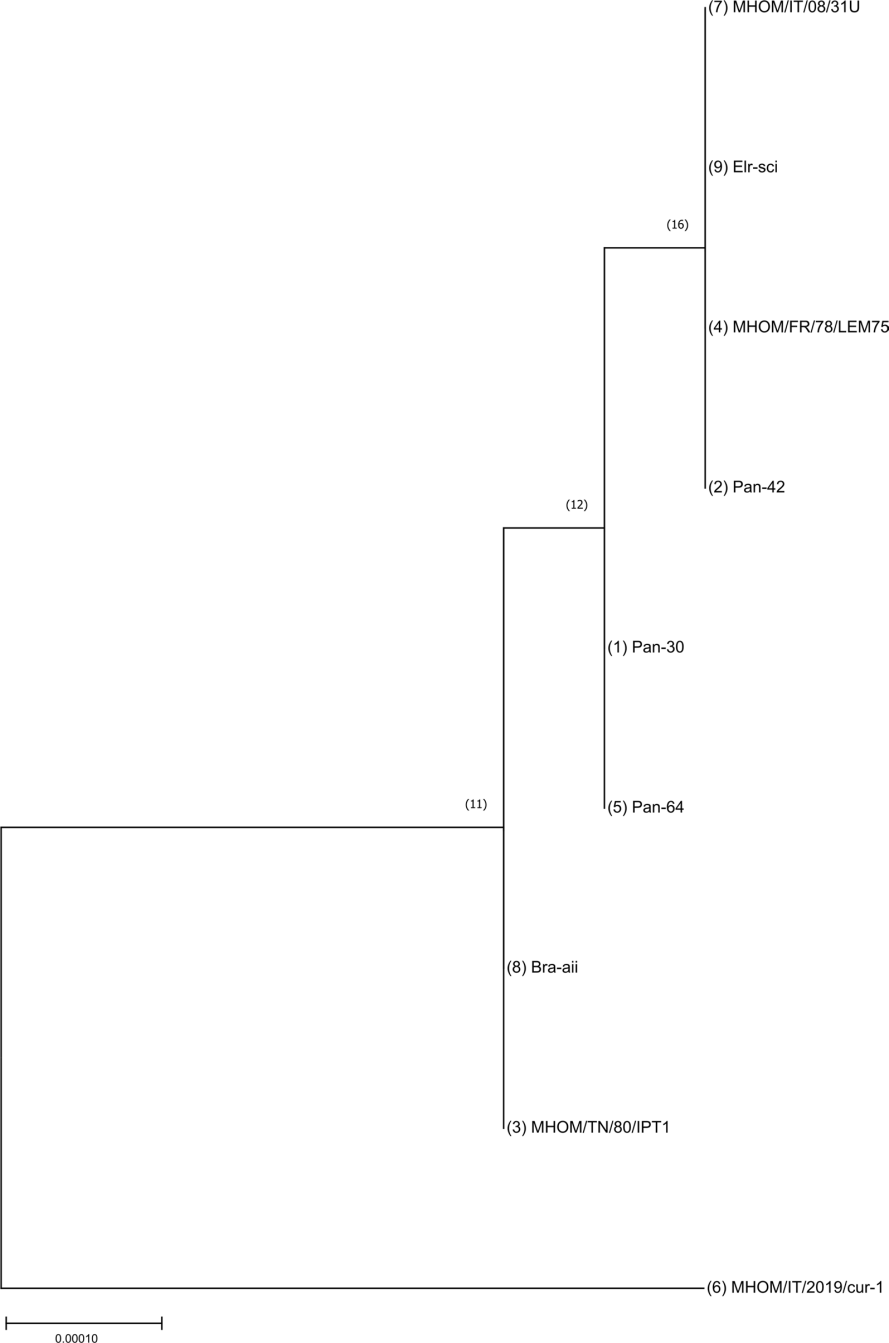
Molecular phylogenetic analysis by the maximum likelihood method. The evolutionary history was inferred by using the maximum likelihood method and the general time reversible model. The tree with the highest log likelihood (− 21365.37) is shown. The percentage of trees in which the associated taxa clustered together is shown next to the branches. Initial tree(s) for the heuristic search were obtained automatically by applying neighbor-joining and BioNJ algorithms to a matrix of pairwise distances estimated using the maximum composite likelihood (MCL) approach, and then selecting the topology with superior log likelihood value

The results of the MLST (Sequence Read Archive [SRA] accession no.: PRJNA911512) showed near-identical sequences (1–3 mismatches compared to reference genome out of 15,488 bases) in all the samples, regardless of geographic origin or host, with the exception of the human isolate from central Italy, MHOM/IT/2019/cur-1, that showed 25 mismatches (Table 3). Specifically, phylogenetic analysis showed that the isolate MHOM/IT/2019/cur-1 clustered independently from the other *L. infantum* samples (Fig. 1). The analysis of MLST results, together with sequences available in Genbank (see Tables 3 and 6 in [10]), enabled confirmation of the polymorphism 390T/G on the ME gene (corresponding to nucleotide 281164 on chromosome 24 on the reference *L. infantum* JPCM5 genome GCA_900500625.2) and of the polymorphism 1831A/G on the GPI gene (corresponding to nucleotide 292809 on chromosome 12 on the reference *L. infantum* JPCM5 genome GCA_900500625.2), as the most informative among all the polymorphisms found (PIC = 0.47 and 0.35, respectively). Based on these results, two HRM-based assays (i.e. qPCR-ME65 and qPCR-GPI88) were developed to rapidly detect those polymorphisms in clinical samples. The first is an update of a previously published assay [10], and the second is a completely novel assay.

**Table 3:** Polymorphisms identified in samples sequenced by multilocus sequence typing, compared to *Leishmania infantum* JPCM5 genome GCA_900500625.2

Protein-coding genes^a^	Sample ID^b^								
	Pan-30	Pan-42	MHOM/TN/80/IPT1	MHOM/FR/78/LEM75	Pan-64	MHOM/IT/2019/Cur-1	MHOM/IT/08/31U	Bra-aii	Elr-sci
Elongation initiation factor 2alpha LINF_030014900									
Spermidine synthase LINF_040010800									
Isocitrate dehydrogenase LINF_100008300						130806C 131265Y 131511T			
Glucose-6-phosphate isomerase LINF_120010600	92519A	292519A	292519A *2292809A*	292519A	292519A *2292809A*	292519A *2292809R*	292519A	292519A *2292809A*	292519A
Inosine-guanine nucleoside hydrolase LINF_140006200						32,800 T			
Nonspecific nucleoside hydrolase LINF_180021400						686,833 G			
Malic enzyme LINF_240012800			*281164G*			280978Y 281103G *281164G* 281281G 281617Y 281693R		*281164G*	
Phosphomannose isomerase LINF_320021600						621512			
Glucose-6-phosphate 1-dehydrogenase LINF_340005700						26839S 26878C 27181C 27947R			
Malate dehydrogenase LINF_340006400						47256R 47877C 48169M 48225G			
Arginase LINF_350019900						571026M			
6-Phosphogluconate dehydrogenase LINF_350038800						1288246C			
Phosphomannomutase LINF_360026300						783228S			
UDP-N-Acetylglucosamine-dolichyl-phosphate N-acetylglucosaminephosphotransferase LINF_360051000									

Italics indicate the position of polymorphisms 1831A/G on the glucose-6-phosphate isomerase gene (nucleotide 292809 on chromosome 12) and polymorphisms 390T/G on the malic enzyme gene (nucleotide 281164 on chromosome 24) on which the high-resolution melting assays have been designed

^a^Fourteen protein-coding genes were selected for the multilocus sequence typing (MLST) assay

^b^See Table 1 for a full description

### Specificity and sensitivity evaluation of the qPCR assay

The new pairs of primers (MEint-F/ME65-R, GPIext-F/GPIext-R and GPI88-F/GPI88-R) were tested by qPCR to evaluate their specificity using DNA from *L. infantum*, *T. cruzi*, human, canine and feline samples. Amplified fragments were analyzed by agarose gel electrophoresis as described in the Methods section. The products obtained were of the expected size, and non-specific amplification was not detected (Additional file 4: Figure S3).

**Fig. 2:**
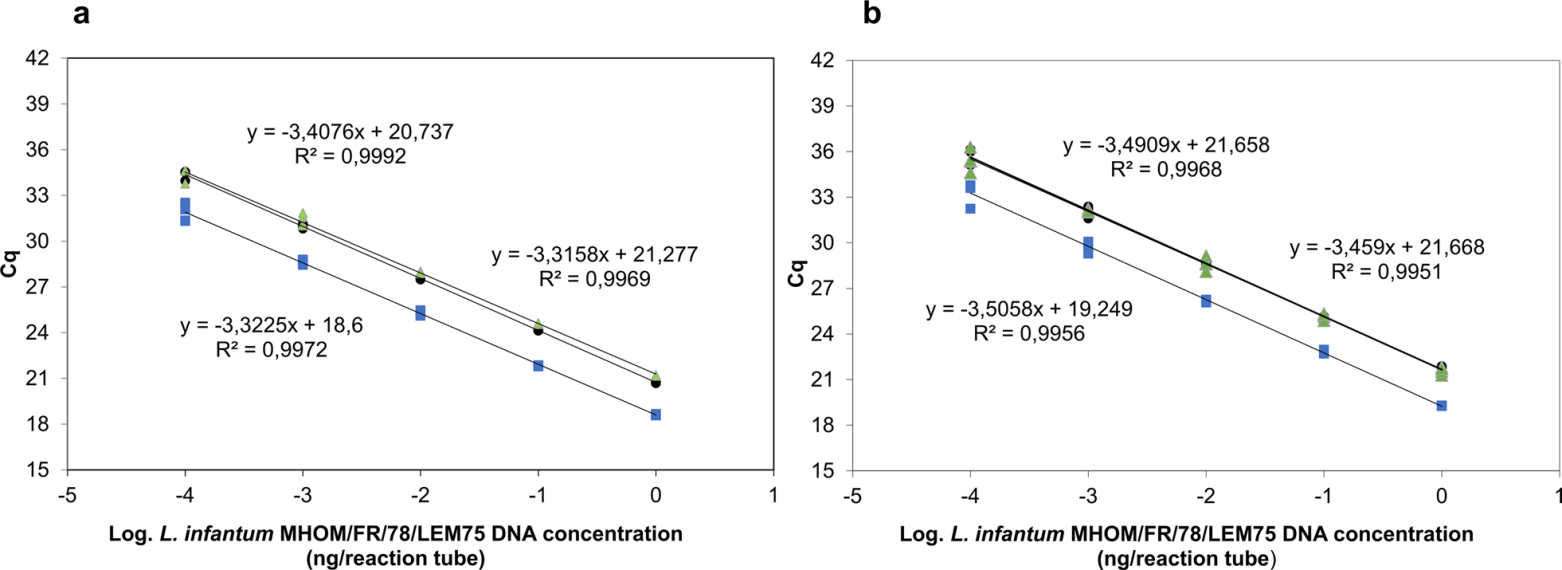
Sensitivity curves of the qPCR-ME65 assay (**a**) and qPCR-GPI88 assay (**b**). The standard curves were spiked with 30 ng of human or canine DNA (upper curves partially overlapping, circles and triangles points, respectively) or non-spiked (lower curve, square points). Each point represents duplicate result of two independent experiments. Cq, Quantification cycle; GPI, glucose-6-phosphate isomerase; ME, malic enzyme; qPCR, quantitative real-time PCR

The sensitivity curves for qPCR ME65 and qPCR GPI88 evidenced a limit of quantification of 1 × 10^−4^ ng of DNA per PCR tube with* R*^2^ > 0.99 (Fig. 2). In the presence of human and canine DNA as background, the quantification cycle (Cq) was slightly delayed, but the linearity and the limit of quantification of the assay remained unchanged.

### Application of HRM assays to *L. infantum* strains

**Fig. 3:**
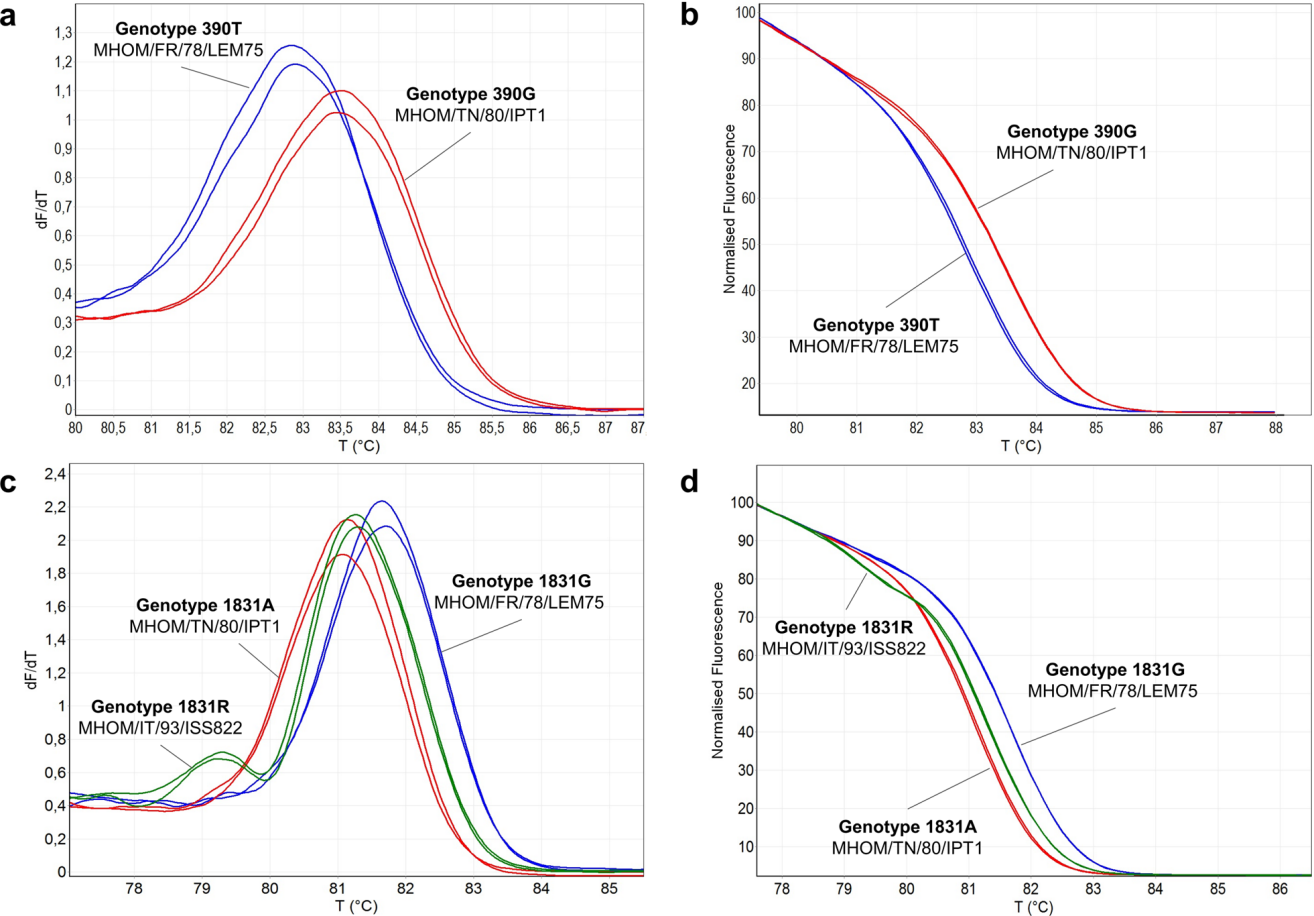
HRM analysis of *Leishmania infantum* reference strains.** a**,** b** qPCR-ME65 HRM negative derivative plots (**a**) and normalized profiles (**b**) were obtained by using DNA from MHOM/TN/80/IPT1 (genotype 390G) and MHOM/FR/78/LEM75 (genotype 390T). **c**,** d** qPCR-GPI88 HRM negative derivative plots (**c**) and normalized profiles (**d**) were obtained by using DNA from MHOM/TN/80/IPT1 (genotype 1831A), MHOM/FR/78/LEM75 (genotype 1831G) and MHOM/IT/93/ISS822 (genotype 1831R). dF/dT, derivative of the intensity of fluorescence at different temperatures; GPI, Glucose-6-phosphate isomerase; HRM, high-resolution melting; ME, malic enzyme; qPCR, quantitative real-time PCR; T, temperature

In order to evaluate the capability of the HRM assays to discriminate the genotypes, the two qPCR assays (qPCR-ME65 and qPCR-GPI88) were first tested on eight *L. infantum* strains (sample nos. 13–20, Table 1) with known sequences. *Leishmania infantum* MHOM/FR/78/LEM75, MHOM/TN/80/IPT1 and MHOM/IT/93/ISS822 strains were selected as references for genotypes 390T/1831G, 390G/1831A and 390T/1831R, respectively. The qPCR and HRM analyses were performed as described in the Methods section, and the results showed that the different genotypes could be differentiated by either HRM curve analysis (negative derivative plot) or HRM normalized profiles (Fig. 3). In particular, the GPI heterozygous strain (1831R) was clearly distinguishable with both the negative derivative plots (showing a typical double peak; Fig. 3c) or with normalized HRM profiles that showed a characteristic shape (Fig. 3d). Comparable results were obtained with the strains MHOM/DZ/82/LIPA59 (390G/1831A), MHOM/ES/81/BCN1 (390G/1831G), MHOM/IT/86/ISS218 (390T/1831G), MHOM/IT/08/31U (390T/1831G) and MHOM/IT/08/49U (390T/1831G) (results not shown).

### Application of HRM assays to *L. infantum* clinical samples/isolates

**Fig. 4:**
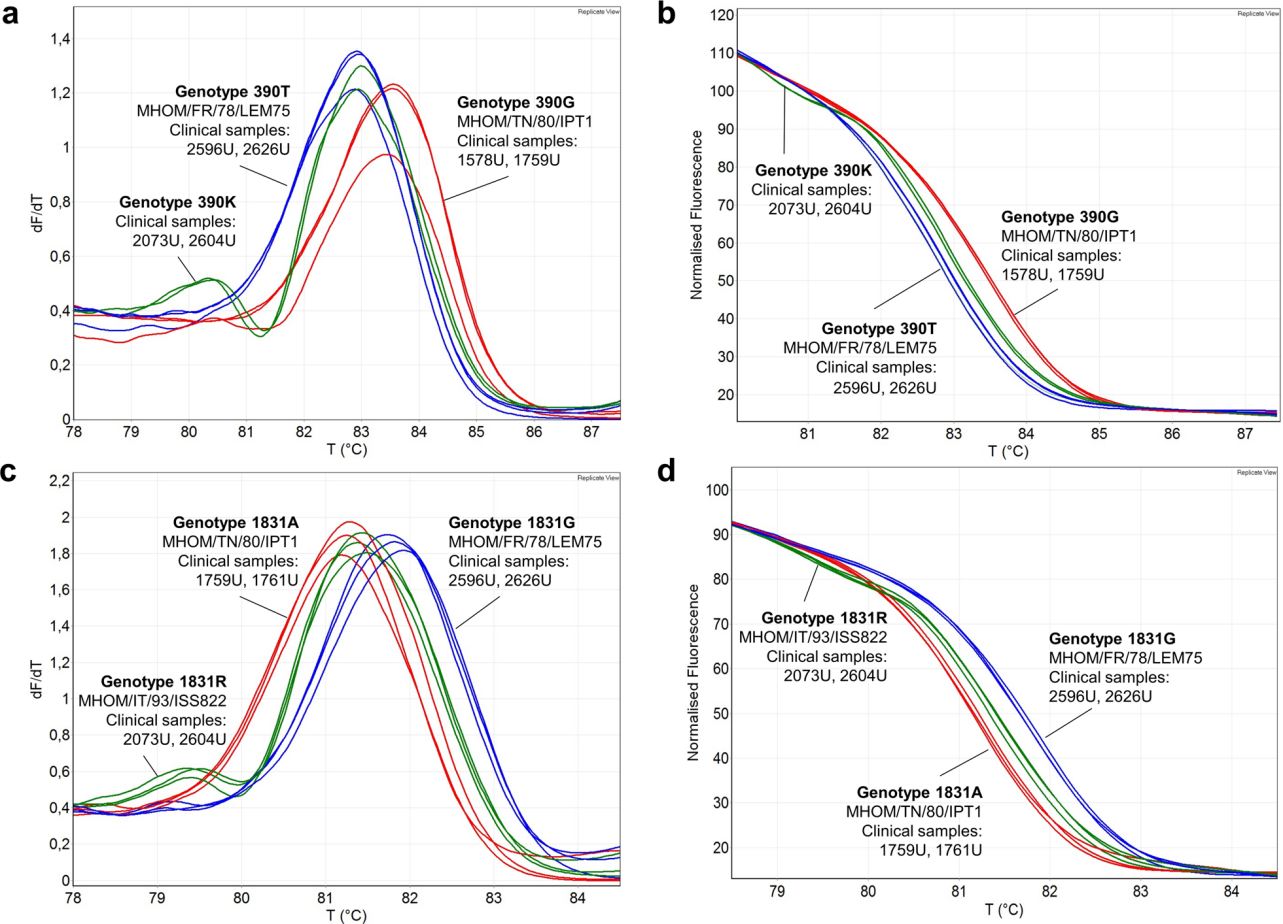
HRM analysis of *Leishmania infantum* clinical samples.** a**,** b** qPCR-ME65 HRM negative derivative plots (**a**) and normalized profiles (**b**) of selected clinical samples. As references, MHOM/TN/ 80/IPT1 (genotype 390G) and MHOM/FR/78/LEM75 (genotype 390 T) strains were included in the assay. **c**,** d** qPCR-GPI88 HRM negative derivative plots (**c**) and normalized profiles (**d**) of selected clinical samples. As references, MHOM/TN/80/IPT1 (genotype 1831A), MHOM/FR/78/LEM75 (genotype 1831G) and MHOM/IT/93/ISS822 (genotype 1831R) strains were included in the assay. dF/dT, derivative of the intensity of fluorescence at different temperatures; HRM, High-resolution melting; GPI, glucose-6-phosphate isomerase; ME, malic enzyme; qPCR, quantitative real-time PCR

Once the potential of the two qPCR assays to discriminate the 390T/G and 1831A/G genotypes in reference strains had been demonstrated, these two assays were tested on nine clinical isolates and 64 human and canine clinical samples from central/southern Italy and Pantelleria Island, with the aim to evaluate the genetic variability of *L. infantum* in these areas by using the HRM-based method. First, the presence of *L. infantum* in clinical samples was confirmed by ITS1-PCR RFLP [18] and/or by qPCRs targeting kinetoplast DNA (kDNA) minicircles [16]. To increase the sensitivity and robustness of the 390T/G and 1831A/G HRM assays on clinical samples, in which the parasite DNA was poorly represented, 15 cycles of the pre-amplification step were introduced using the external primers pair, as described in the Methods section. Subsequently, 1 µl of the reaction was used as template for the qPCR assays. Genotypes of 61 and 53 samples were distinguished by the qPCR-ME65 and qPCR-GPI88 assays, respectively, in the 64 clinical samples available, showing a sensitivity of 95.3% and 82.8%. Notably, the sensitivity reached 100% on clinical isolates (9 out of 9 with both assays). An example of negative derivative plots and normalized HRM profiles obtained with the clinical samples is shown in Fig. 4. Interestingly, the human samples 2073U and 2604U showed a normalized HRM profile and an HRM double peak attributable to the heterozygote genotype for both ME and GPI genes (390K/1831R) (Fig. 4). Because of the absence of a heterozygote reference strain for the ME gene, these amplicons were sequenced as described above. The electropherograms showed a double peak at position 390 compatible with heterozygosity (390K) (Additional file 5: Figure S4).

### HRM temperature analyses

**Fig. 5:**
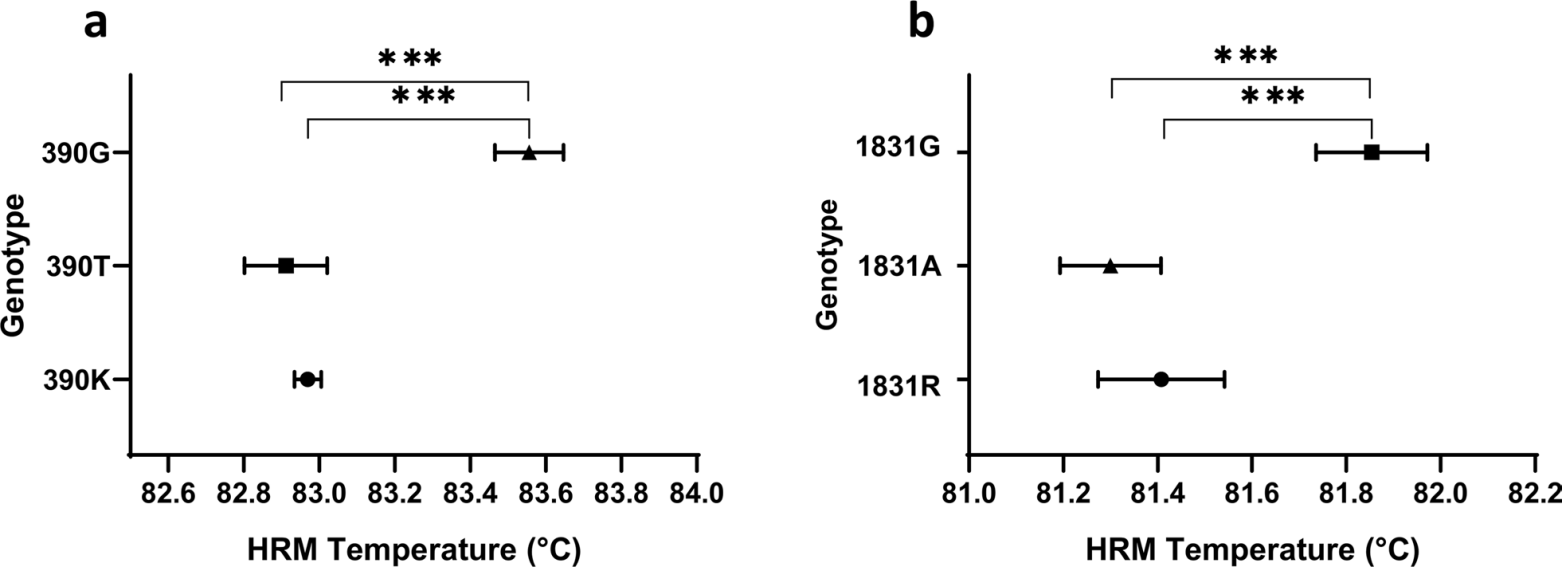
HRM temperatures of *Leishmania infantum* samples. **a** HRM temperatures of amplicons obtained by qPCR-ME65, **b** HRM temperatures of amplicons obtained by qPCR-GPI88. Temperatures represent the mean ± SD of the technical replicates of at least two independent experiments. Asterisks indicate a significant difference at ***p < 0.001, Kruskal–Wallis followed by Dunn's multiple comparisons test. HRM, High-resolution melting; GPI, glucose-6-phosphate isomerase; ME, malic enzyme; qPCR, quantitative real-time PCR

The genotype discrimination power of the qPCR-ME65 and qPCR-GPI88 assays was also evaluated by the analyzing the HRM temperatures of all *L. infantum* samples. The qPCR-ME65 assay provided evidence of a statistically significant difference between the mean (± SD) melting temperature (Tm) of genotype 390G (83.56 °C ± 0.09 °C) and that of genotypes 390T or 390K (82.91 °C ± 0.11 °C and 82.97 °C ± 0.04 °C, respectively) (Kruskal–Wallis followed by Dunn's multiple comparisons test,* P* < 0.001) (Fig. 5a), confirming the applicability of the method for clinical samples. In the same way, the mean Tm (± SD) in the qPCR-GPI88 assay allowed the samples with genotype 1831G (81.85 °C ± 0.12 °C) to be distinguished from those with genotype 1831A or 1831R (81.30 °C ± 0.11 °C and 81.41 °C ± 0.14 °C, respectively) (Kruskal–Wallis followed by Dunn's multiple comparisons test,* P* < 0.001) (Fig. 5b). However, the Tm of the samples with heterozygote amplicons (i.e. 390K and 1831R) showed a partial overlapping with the Tm of samples with genotype 390T or 1831A, respectively; hence the two genotypes were not distinguishable exclusively based on the Tm. Nevertheless, this drawback can be overcome by the presence of the second HRM peak. Although the analysis of this second peak required the fluorescence threshold to be lowered, the peak was distinguishable from the background and showed a mean Tm (± SD) of 80.39 °C ± 0.08 °C and 79.40 °C ± 0.14 °C for the 390K and 1831R amplicons, respectively (Fig. 4a, c). This feature of heterozygote samples was confirmed by the HRM normalized profiles (Fig. 4b, d).

### Confirmation of HRM assay results by sequencing

In order to confirm the genotype assigned by the Rotorgene software based on the HRM analysis, we first purified the amplification products of the reference strains and many clinical samples and then sequenced these as described in Methods. Other sequences were obtained by the MLST panel (SRA accession no.: PRJNA911512) or from our previous publication [10]. The presence of two different overlapping peaks at the same nucleotide position was associated with a heterozygote genotype. In summary, 43 and 53 sequences encompassing the polymorphisms in the ME and GPI genes were available. The results showed a 100% correlation with the genotype determination based on the HRM assays. Additional file 5: Figure S4 and Additional file 6: Figure S5 show electropherograms of selected clinical amplicons obtained with qPCR-MEint and qPCR-GPIext, respectively.

### Genotype identification

**Fig. 6:**
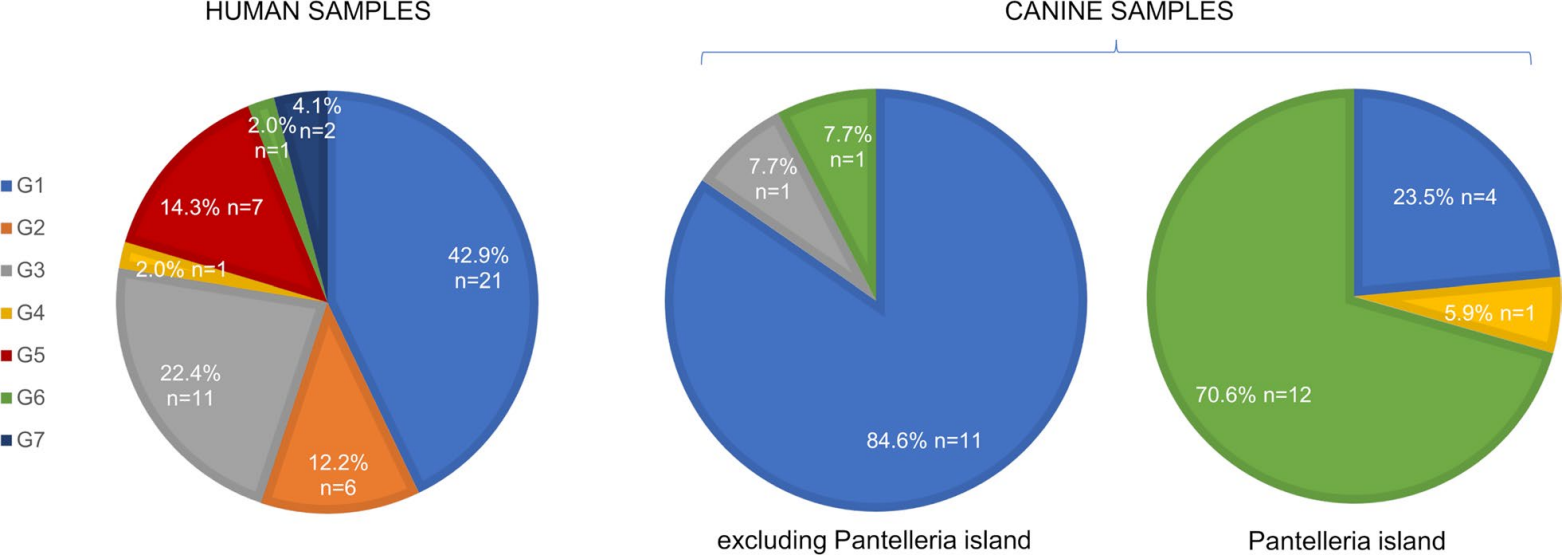
Genotype distribution in human and canine samples. Human samples (*n* = 49) include all seven genotypes with a marked prevalence of the G1 genotype (42.9%), followed by the G3 (22.4%) and G5 genotypes (14.3%). Canine samples from central/southern Italy (*n* = 13) showed a marked prevalence of the G1 genotype (84.6%), while canine samples from Pantelleria island (n = 17) showed a prevalence of the G6 genotype (70.6%).

Based on the results of the simultaneous analysis of the two polymorphisms, we were able to assign seven different genotypes (G1-G7) in 82 of 93 samples. Among these seven genotypes, genotype 1 (G1) was the most highly represented (38 out of 82 samples; prevalence of 46.3%) (Table 4). The genotyping results for each *L. infantum* strain, clinical sample and clinical isolate are summarized in Table 5. It is noteworthy that the G1 genotype characterized 12 out of 14 (85.7%) *L. infantum* strains/isolates belonging to MON-1 and MON-72 zymodemes. In contrast, the G1 genotype was not found in any of the non-MON-1/MON-72 zymodemes (Table 5). Excluding samples from Pantelleria Island, G1 was confirmed as the most frequent genotype in both human (prevalence of 42.9%) and canine (prevalence of 84.6%) populations; notably, human samples showed more genetic variability compared to those of dogs (Fig. 6). However, in the insular context of Pantelleria Island, the situation of canine samples is completely reversed: 12 out of 17 samples showed genotype 6 (G6) (390T/1831A; prevalence of 70.6%) and the G1 genotype had a prevalence of only 23.5% (Fig. 6). Canine sample Els-mai, originating from the veterinary clinic "Santa Teresa" in Fano (central Italy) was an unusual case in which the two conjunctival swabs belonged to two different genotypes (Els-mai^a^: G6; Els-mai^b^: G1). Moreover, two human samples (i.e. 2073U, 2604U) showed heterozygosity in both ME and GPI polymorphisms (G7).

**Table 4:** Genotypes identified in this study and percentage distribution in all samples

Genotype	ME	GPI	Number of samples	% Distribution
G1	390T	1831G	38	46.3
G2	390G	1831G	6	7.3
G3	390G	1831A	12	14.6
G4	390T	1831R	2	2.4
G5	390G	1831R	8	9.8
G6	390T	1831A	14	17.1
G7	390K	1831R	2	2.4

*GPI* Glucose-6-phosphate isomerase,* ME* malic enzyme

**Table 5:** Summary of genotyping results of strains and clinical samples/isolates analyzed in this study

Sample no.	Sample ID	Type of sample (zymodeme)	ME sequence	qPCR-ME65	GPI sequence	qPCR-GPI88	Genotype
1	MCAN/ES/98/LLM-724	Reference strain (MON-1)	390T^1^	n.a.	1831G^1^	n.a.	1
2	MHOM/PT/2000/IMT260	Reference strain (MON-1)	390T^1^	n.a.	1831G^1^	n.a.	1
3	MHOM/ES/1986/BCN16	Reference strain (MON-1)	390T^1^	n.a.	1831G^1^	n.a.	1
4	MHOM/FR/1997/LSL29	Reference strain (MON-1)	390T^1^	n.a.	1831G^1^	n.a.	1
5	MHOM/ES/1993/PM1	Reference strain (MON-1)	390T^1^	n.a.	1831G^1^	n.a.	1
6	MHOM/FR/1995/LPN114	Reference strain (MON-1)	390T^1^	n.a.	1831G^1^	n.a.	1
7	MHOM/FR/1996/LEM3249	Reference strain (MON-29)	390G^1^	n.a.	1831G^1^	n.a.	2
8	MHOM/ES/1991/LEM2298	Reference strain (MON-183)	390G^1^	n.a.	1831G^1^	n.a.	2
9	MHOM/ES/1988/LLM175	Reference strain (MON-198)	390G^1^	n.a.	1831G^1^	n.a.	2
10	MHOM/IT/1994/ISS1036	Reference strain (MON-228)	390G^1^	n.a.	1831G^1^	n.a.	2
11	MHOM/ES/1992/LLM373	Reference strain (MON-199)	390G^1^	n.a.	1831R^1^	n.a.	5
12	MHOM/MT/1985/BUCK	Reference strain (MON-78)	390G^1^	n.a.	1831A^1^	n.a.	3
13	MHOM/FR/78/LEM75	Reference strain (MON-1)	390T^2^	390T	1831G^2^	1831G	1
14	MHOM/TN/80/IPT1	Reference strain (MON-1)	390G^2^	390G	1831A^2^	1831A	3
15	MHOM/DZ/82/LIPA59	Reference strain (MON-24)	390G^1^	390G	1831A^3^	1831A	3
16	MHOM/ES/81/BCN1	Reference strain (MON-29)	390G^1^	390G	1831G^3^	1831G	2
17	MHOM/IT/86/ISS218	Reference strain (MON-72)	390T^1^	390T	1831G^3^	1831G	1
18	MHOM/IT/93/ISS822	Reference strain (MON-201)	390T^1^	390T	1831R^3^	1831R	4
19	MHOM/IT/08/31U	Reference strain (MON-1)	390T^2^	390T	1831G^2^	1831G	1
20	MHOM/IT/08/49U	Reference strain (MON-1)	390T^1^	390T	n.a.	1831G	1
21	Pan-30	Clinical isolate	390T^2^	390T	1831A^2^	1831A	6
22	Pan-42	Clinical isolate	390T^2^	390T	1831G^2^	1831G	1
23	Pan-64	Clinical isolate	390T^2^	390T	1831A^2^	1831A	6
24	10816	Clinical isolate (MON-1)	390T^1^	390T	n.a.	1831G	1
25	V2921	Clinical isolate (MON-1)	n.a.	390G	1831R^3^	1831R	5
26	791	Clinical isolate (MON-1)	390T^1^	390T	n.a	1831G	1
27	MHOM/IT/2019/cur-1	Clinical isolate	390G^2^	390G	1831R^2^	1831R	5
28	Elr-sci	Clinical isolate	390T^2^	390T	1831G^2^	1831G	1
29	Bra-aii	Clinical isolate	390G^2^	390G	1831A^2^	1831A	3
30	Plo-roi	Clinical sample	n.a.	390T	n.a.	1831G	1
31	Aro-sai	Clinical sample	390T^3^	390T	1831G^3^	1831G	1
32	Els-mai^a^	Clinical sample	n.a.	390T	1831A^3^	1831A	6
33	Els-mai^b^	Clinical sample	n.a.	390T	1831G^3^	1831G	1
34	Toy-gai	Clinical sample	n.a.	390T	n.a.	1831G	1
35	Koa-cro	Clinical sample	n.a.	390T	n.a.	1831G	1
36	Zeo-sci	Clinical sample	n.a.	390T	1831G^3^	1831G	1
37	Gia-spi	Clinical sample	390T^1^	390T	1831G^3^	1831G	1
38	Grg-rao	Clinical sample	390T^1^	390T	n.a.	1831G	1
39	Vea-fri	Clinical sample	390T^1^	390T	n.a.	n.d.	
40	Jon-doe	Clinical sample	n.a.	390T	1831G^3^	1831G	1
41	Pan-1	Clinical sample	n.a.	390T	1831A^3^	1831A	6
42	Pan-2	Clinical sample	n.a.	390T	n.a.	n.d.	
43	Pan-4	Clinical sample	n.a.	390T	1831R^3^	1831R	4
44	Pan-5	Clinical sample	n.a.	390T	1831A^3^	1831A	6
45	Pan-6	Clinical sample	n.a.	390T	1831A^3^	1831A	6
46	Pan-9	Clinical sample	n.a.	390T	1831A^3^	1831A	6
47	Pan-10	Clinical sample	n.a.	390T	n.a.	1831A	6
48	Pan-11	Clinical sample	n.a.	390T	n.a.	n.d.	
49	Pan-12	Clinical sample	n.a.	390T	n.a.	1831A	6
50	Pan-13	Clinical sample	n.a.	390T	n.a.	n.d.	
51	Pan-14	Clinical sample	n.a.	390T	1831G^3^	1831G	1
52	Pan-15	Clinical sample	n.a.	390T	n.a.	n.d.	
53	Pan-16	Clinical sample	n.a.	390T	n.a.	1831A	6
54	Pan-21	Clinical sample	n.a.	390T	1831G^3^	1831G	1
55	Pan-22	Clinical sample	n.a.	390T	1831G^3^	1831G	1
56	Pan-24	Clinical sample	n.a.	390T	n.a.	n.d.	
57	Pan-25	Clinical sample	n.a.	n.d.	n.a.	n.d.	
58	Pan-26	Clinical sample	390T^3^	390T	1831A^3^	1831A	6
59	Pan-27	Clinical sample	n.a.	390T	1831A^3^	1831A	6
60	Pan-28	Clinical sample	n.a.	390T	1831A^3^	1831A	6
61	Psalb	Clinical sample	390T^1^	390T	1831G^3^	1831G	1
62	Dae-dio	Clinical sample	390G^3^	390G	1831R^3^	1831R	5
63	Mao-pai	Clinical sample	n.a.	390G	1831R^3^	1831R	5
64	Gae-bea	Clinical sample	390G^3^	390G	1831R^3^	1831R	5
65	Kua-asn	Clinical sample	n.a.	390G	n.a.	1831A	3
66	1038U	Clinical sample	n.a.	390T	n.a.	1831G	1
67	1522U	Clinical sample	n.a.	390T	n.a.	1831G	1
68	1536U	Clinical sample	n.a.	390T	n.a.	1831G	1
69	1538U	Clinical sample	n.a.	390T	n.a.	1831G	1
70	1578U	Clinical sample	n.a.	390G	n.a.	1831G	2
71	1758U	Clinical sample	390G^3^	390G	n.a.	1831R	5
72	1759U	Clinical sample	390G^3^	390G	1831A^3^	1831A	3
73	1761U	Clinical sample	390G^3^	390G	1831A^3^	1831A	3
74	1810U	Clinical sample	n.a.	390G	n.a.	n.d.	
75	2000U	Clinical sample	n.a.	n.d.	n.a.	n.d.	
76	2068U	Clinical sample	390T^3^	390T	1831G^3^	1831G	1
77	2073U	Clinical sample	390K^3^	390K	1831R^3^	1831R	7
78	2579U	Clinical sample	390T^3^	390T	1831A^3^	1831A	6
79	2596U	Clinical sample	390T^3^	390T	1831G^3^	1831G	1
80	2602U	Clinical sample	390G^3^	390G	1831A^3^	1831A	3
81	2604U	Clinical sample	390K^3^	390K	1831R^3^	1831R	7
82	2618U	Clinical sample	390T^3^	390T	1831G^3^	1831G	1
83	2619U	Clinical sample	390T^3^	390T	1831G^3^	1831G	1
84	2626U	Clinical sample	390T^3^	390T	1831G^3^	1831G	1
85	2629U	Clinical sample	390G^3^	390G	1831A^3^	1831A	3
86	2632U	Clinical sample	n.a.	n.d.	n.a.	n.d.	
87	2647U	Clinical sample	390G^3^	390G	1831A^3^	1831A	3
88	2652U	Clinical sample	390T^3^	390T	1831G^3^	1831G	1
89	2660U	Clinical sample	390G^3^	390G	1831A^3^	1831A	3
90	2668U	Clinical sample	n.a.	390T	n.a.	n.d.	
91	2669U	Clinical sample	390T^3^	390T	1831G^3^	1831G	1
92	2746U	Clinical sample	390G^3^	390G	1831R^3^	1831R	5
93	2897U	Clinical sample	390G^3^	390G	1831A^3^	1831A	3

*GPI* Glucose-6-phosphate isomerase, *ME* malic enzyme,* n.a.* not available;* n.d.* not detected,* qPCR* quantitative real-time PCR

Els-mai^a^ was a left conjunctival swab; Els-mai^b^ was a right conjunctival swab

^1^Sequences published in Ceccarelli et al*.* [10]

^2^Sequences obtained by MLST

^3^Sanger sequences obtained by ABI PRISM 310 Genetic Analyzer

## Discussion

The taxonomy of the *Leishmania* genus is very complex and has been revised several times over the years according to the biochemical and biological characteristics of the parasites [26]. In the present study, we focused on *L. infantum* species, the main causative agent of CanL and human VL and CL. *Leishmania* typing at the species and strain level is important for epidemiological studies [27], for the identification of new reservoirs and to predict the clinical course of infection, particularly in humans (dermotropic and viscerotropic strains) [28]. The reference technique for typing members of genus *Leishmania* is the MLEE method [1], which is based on the electrophoretic mobility of various enzymes obtained from the promastigotes. Based on this technique, *Leishmania* species are classified in zymodemes (also referred to as MON). In Italy, MON-1 and MON-72 are the most highly represented *L. infantum* zymodemes in infected dogs, while human infections are caused by a more heterogeneous zymodeme population [27], suggesting that dogs are not the only reservoirs of infection and emphasizing the importance of epidemiological studies. A recent work by Castelli et al. [29] conducted in Sicily (Italy) on *Leishmania* isolates from humans and dogs revealed that 71 out of 78 samples (91%) were MON-1, which was confirmed as the predominant zymodeme in the Mediterranean area; the remaining seven samples (9%) were non-MON-1. In particular, the non-MON-1 strains were isolated from humans. Moreover, an increasingly number of studies are investigating the role of other mammalians as *Leishmania* reservoirs, such as hares [30], rabbits [31], wolves [32] and, in particular, domestic cats [33]. In this context, an approach that allows for rapid parasite characterization could be useful for epidemiological studies. However, the MLEE technique is associated with a number of limitations, including the need for parasite cultivation. As alternative to MLEE, in addition to the MLST and MLMT methods, other approaches have been attempted to characterize genetic diversity in *L. infantum* populations. In particular, recent studies have exploited the SNP on kDNA minicircles to identify different *L. infantum* genotypes in the Mediterranean area [34, 35]. This approach was based on PCR amplification of a minicircle region followed by RFLP analysis or DNA sequencing, then by in silico RFLP.

The aim of the present study was to find an alternative, fast and inexpensive screening method to explore genetic variability of *L. infantum* circulating in Mediterranean region. We therefore focused on HRM-based monitoring of SNPs found in metabolic enzymes used in the MLEE approach. To identify polymorphisms useful for rapid *L. infantum* typing, we first designed a custom MLST panel and then sequenced nine *L. infantum* strains and clinical isolates. Based on MLST panel results, an informative polymorphism on GPI genes (1831A/G) was identified and selected to develop a qPCR-HRM-based assay for clinical sample screening, to be used in association with an updated genotyping method initially developed and applied by Ceccarelli et al*.* [10] on the ME gene. Remarkably, due to the pre-amplification step, it was possible to apply this method to clinical samples without parasite isolation. This aspect represents an important advantage if compared to other biomolecular approaches for *Leishmania* typing performed on clinical strains and isolates [36, 37].

Notably, in our previous work, we showed that the 390T polymorphism on the ME gene was associated with zymodemes MON-1, MON-72 and MON-201 [10]. In the present study, with the simultaneous use of the two polymorphisms, we were able to distinguish the strain MHOM/IT/93/ISS822 (MON-201) from MON-1 and MON-72 exploiting the heterozygosity found in position 1831 of the GPI gene, as also confirmed by sequencing (Additional file 6: Figure S5). Heterozygosity was also found to be present in the clinical isolates V2921 and MHOM/IT/2019/cur-1 and in eight clinical samples. The heterozygosity is not surprising since it has been reported previously in other metabolic enzymes of the *Leishmania donovani* complex [37], in association with the geographical origin of the parasite [38]. However, the detection of heterozygosity could present some limitations to the assay. In fact, *Leishmania* spp. is a parasite with a constitutive aneuploidy [39, 40] and it is possible that our HRM approach can identify the heterozygosity only if it is close to 50%. Nevertheless, this new HRM-based genotyping method, using only two molecular targets, was able to distinguish seven different genotypes (named G1-G7). A univocal correlation between the MLEE and HRM-based genotyping assays is not possible since the two approaches are different. However, the results of this study showed that genotype G1 (the most prevalent genotype) has a strong correlation (although not univocal) with zymodemes MON-1 and MON-72. In fact, 85.7% of the MON-1 and MON-72 stains/isolates having available ME and GPI sequences were assigned to the G1 genotype. The only exceptions were the reference strains MHOM/TN/80/IPT1 and V2921, which, despite being MON-1, were assigned to the G3 and G5 genotypes, respectively. It is possible that the geographical origin of strain MHOM/TN/80/IPT1 (a Tunisian strain) or to the host (V2921 was isolated from a marten) led to these differences. Despite the increase in discrimination power in the method reported in the present study compared to that reported in our previous publication [10], no genotypic differences were found between the MON-1 and MON-72 zymodemes. However, this may not represent an issue. In fact, MON-1 and MON- 72 are the most highly represented zymodemes in dogs [29, 41]; therefore, the identification of genotypes different from G1 could be useful to study and understand the exact role of dogs in the transmission of the pathogen and to identify other reservoirs of infection for humans. With the exclusion of samples from Pantelleria Island, 42.9% and 84.6% of human and canine clinical samples/isolates were assigned to the G1 genotype. Based on the correlation between G1 and MON-1, the results can be considered to be in agreement with published data showing that MON-1 is the predominant zymodeme in the Mediterranean area [5]. The fact that the percentage of the G1 genotype in human samples was just over half that in canine samples seems to confirm the greater genetic variability of parasites in humans compared to dogs, which is explainable by considering the role of other mammals as reservoirs of infection, as mentioned above. Notably, different genotype distributions between humans and dogs have also been described previously in a small geographical area using different genetic markers [34], thereby reinforcing the possibility that *L. infantum* circulating in humans may rely on multiple reservoirs. However, since *L. infantum* genotype comparisons among different studies are still complicated by the number of methods and sequences used, the unification and standardization of molecular markers would be needed for a better understanding of the parasite epidemiology.

Regarding samples from Pantelleria Island, it should be noted that this island is characterized by ideal conditions to study leishmaniasis in a defined population of animals and in a circumscribed and central territory of the Mediterranean basin. Previous studies reported a Leishmaniasis prevalence of 27% in the dog population of the island, which is in line with the average prevalence in the Sicily region, indicative of an active circulation of the parasite in the island [12, 42]. In this area, only 23.5% of canine clinical isolates/samples were associated with the G1 genotype while 70.6% were identified as the G6 genotype (390T/1831A). The enrichment of the G6 genotype in samples from Pantelleria island, compared to the genotypes in other samples from the Italian peninsula, could be explained by the natural isolation of Pantelleria Island that resulted in limited mixing of the genetic groups.

In addition to all of the above-mentioned considerations, interesting results have emerged from some clinical samples. For example, Bra-aii (G3) clusters with MHOM/TN/80/IPT1 in the MLST panel suggesting the possibility that it is a MON-1, confirming the genotypic heterogeneity within the MON-1 zymodeme, as described in other works [43]. Moreover, we obtained two different genotypes from left and right conjunctival swabs of canine clinical sample Els-mai (G1 and G6, respectively), a result that could be explained by a possible co-infection with two different strains of the pathogen. Interesting data was also obtained from two human clinical samples (i.e. 2073U, 2604U) that belong to G7; these were the only samples that were heterozygous for the ME gene.

On the whole, the results obtained, confirmed by PCR product sequencing, have shown that our HRM approach is robust and applicable for clinical sample genotyping, as well as for epidemiological studies. Although using only two SNPs could be a limitation for genotyping purposes due to low discriminatory power, this approach allowed the rapid identification of seven different genotypes, with the major advantage of being able to work on clinical samples with affordable reagents and without the need for parasite isolation.

## Conclusions

A total of nine *L. infantum* isolates/strains have been sequenced by an MLST panel covering 14 metabolic enzymes. The analysis of these sequences and of sequences available in the Genbank database allowed us to identify two informative polymorphisms exploitable for differentiating *L. infantum* genotypes in samples from the Mediterranean basin (390T/G and 1831A/G, in the ME and GPI genes, respectively). Two HRM-based assays were developed to differentiate these genotypes. The application of this technique on nine clinical isolates and 64 clinical samples without parasite isolation allowed us to identify seven different genotypes. Moreover, we found a correlation between genotype G1 (390T/1831G) and zymodemes MON-1 and MON-72, allowing rapid identification of the most common *L. infantum* zymodemes. This is confirmed in our study, where G1 represented 42.9% and 84.6% of human and canine clinical samples/isolates, respectively. Interestingly, this percentage changed in Pantelleria island, where the prevalence of G1 was only 23.5% while that of G6 was 70.6%.

In conclusion, this approach could find application as a rapid and inexpensive screening tool for the characterization of *L. infantum* clinical isolates in epidemiological studies, for identifying new reservoirs of infection and for investigating the genetic variability of *L. infantum*.

### Supplementary Information

**Additional file 1: Table S1.** Genes and genomic coordinates of the MLST panel designed with Ion AmpliSeq™ designer using *L. infantum* JPCM5 genome assembly GCA_900180445 as reference.

**Additional file 2: Figure S1.** Sequence of *L. infantum* MHOM/FR/78/LEM75 (DQ449701.1) ME gene and primers used for PCR-MEint and PCR-ME65. PCR-MEint primers are in bold, ME65-R primer is underlined. MEint-F is in common for both qPCR assays. Polymorphic position (390T/G) is boxed.

**Additional file 3: Figure S2.** Sequence of *L. infantum* MHOM/FR/78/LEM75 (AJ620617.1) GPI gene and primers used for PCR-GPIext and PCR-GPI88. PCR-GPIext primers are underlined, PCR-GPI88 primers are in bold. The polymorphic position (1831A/G) is boxed.

**Additional file 4: Figure S3.** Specificity evaluation of primers GPIext-F/GPIext-R (**a**), MEint-F/ME65-R (**b**) and GPI88-F/GPI88-R (**c**). M, marker 100-bp DNA ladder; LEM75, MHOM/FR/78/LEM75; IPT1, MHOM/TN/80/IPT1; H, human DNA; D, dog DNA; C, cat DNA; T, *Trypanosoma cruzi* DNA; NTC, no template control.

**Additional file 5: Figure S4.** Electropherograms of clinical samples 2073U and 2604U obtained with the qPCR-MEint compared with reference strains. The arrows evidence the diagnostic SNP in position 390.

**Additional file 6: Figure S5.** Electropherograms of selected amplicons obtained with the qPCR-GPIext. The arrows indicate the position 1831 where a diagnostic SNP was found. In particular, the heterozygosis of MHOM/IT/93/ISS822 strain and clinical isolate V2921 is evident. Moreover, the electropherograms demonstrate different results in left and right conjunctival swabs of canine clinical sample Els-mai. Els-mai^a^, Left conjunctival swab; Els-mai^b^, right conjunctival swab.

## Data Availability

The MLST data presented in the study are deposited in the Sequence Read Archive (SRA) of National Center for Biotechnology Information (NCBI) repository, and accession number (SRA accession number) is PRJNA911512.

## Abbreviations

CanL: Canine leishmaniasis
CL: Cutaneous leishmaniasis
cPCR: Conventional PCR
Cq: Quantification cycle
GPI: Glucose-6-phosphate isomerase
HRM: High-resolution melting
kDNA: Kinetoplast DNA
ME: Malic enzyme
MLEE: Multilocus enzyme electrophoresis
MLMT: Multilocus microsatellite typing
MLST: Multilocus sequence typing
NGS: Next-generation sequencing
RFLP: Restriction fragment length polymorphism
qPCR: Quantitative real-time PCR
SNPs: Single nucleotide polymorphisms
Tm: Melting temperature
VL: Visceral leishmaniasis
