# Supplementary material for: High-resolution melting (HRM)-based detection of polymorphisms in the malic enzyme and glucose-6-phosphate isomerase genes for Leishmania infantum genotyping
Source: Parasit Vectors. 2023 Aug 14;16:282. doi: 10.1186/s13071-023-05878-y (PMC10426199; doi:10.1186/s13071-023-05878-y)
Supplement: Supplementary file 1 — Additional file 1: Table S1. Genes and genomic coordinates of the MLST panel designed with Ion AmpliSeq™ designer using L. infantum JPCM5 genome assembly GCA_900180445 as reference. [file 13071_2023_5878_MOESM1_ESM.docx]

**Table S1** Genes and genomic coordinates of the MLST panel designed with Ion AmpliSeq™ designer using *L. infantum* JPCM5 genome assembly GCA_900180445 as reference

| **Name** | **gene ID** | **Chr-Start** | **Chr-End** | **Num Amplicons** | **Total Bases** | **Covered Bases** | **Missed Bases** | **Overall Coverage** |
| --- | --- | --- | --- | --- | --- | --- | --- | --- |
| Elongation initiation factor 2alpha | LINF_030014900 | 386699 | 387940 | 7 | 1241 | 1241 | 0 | 1 |
| Spermidine synthase1 | LINF_040010800 | 235535 | 236437 | 6 | 902 | 902 | 0 | 1 |
| Isocitrate dehydrogenase | LINF_100008300 | 130500 | 131500 | 7 | 1000 | 1000 | 0 | 1 |
| Glucose-6-phosphate isomerase | LINF_120010600 | 291650 | 292850 | 7 | 1200 | 1165 | 35 | 0.971 |
| inosine-guanine nucleoside hydrolase | LINF_140006200 | 31980 | 33040 | 6 | 1060 | 1060 | 0 | 1 |
| nonspecific nucleoside hydrolase | LINF_180021400 | 686250 | 687250 | 6 | 1000 | 864 | 136 | 0.864 |
| Malic enzyme | LINF_240012800 | 280800 | 282400 | 9 | 1600 | 1500 | 100 | 0.938 |
| phosphomannose isomerase | LINF_320021600 | 621550 | 622150 | 4 | 600 | 600 | 0 | 1 |
| glucose-6-phosphate 1-dehydrogenase | LINF_340005700 | 26850 | 28050 | 7 | 1200 | 1200 | 0 | 1 |
| Malate dehydrogenase | LINF_340006400 | 47355 | 48308 | 6 | 953 | 930 | 23 | 0.976 |
| Arginase | LINF_350019900 | 570876 | 571865 | 7 | 989 | 989 | 0 | 1 |
| 6-phosphogluconate dehydrogenase | LINF_350038800 | 1288100 | 1288800 | 4 | 700 | 513 | 187 | 0.733 |
| Phosphomannomutase | LINF_360026300 | 782734 | 783477 | 4 | 743 | 743 | 0 | 1 |
| UDP-N-acetylglucosamine-dolichyl-phosphate N-acetylglucosaminephosphotransferase | LINF_360051000 | 1632000 | 1633300 | 8 | 1300 | 1300 | 0 | 1 |
