# Supplementary material for: High-resolution melting (HRM)-based detection of polymorphisms in the malic enzyme and glucose-6-phosphate isomerase genes for Leishmania infantum genotyping
Source: Parasit Vectors. 2023 Aug 14;16:282. doi: 10.1186/s13071-023-05878-y (PMC10426199; doi:10.1186/s13071-023-05878-y)
Supplement: Supplementary file 2 — Additional file 2: Figure S1. Sequence of L. infantum MHOM/FR/78/LEM75 (DQ449701.1) ME gene and primers used for PCR-MEint and PCR-ME65. PCR-MEint primers are in bold, ME65-R primer is underlined. MEint-F is in common for both qPCR assays. Polymorphic position (390T/G) is boxed. [file 13071_2023_5878_MOESM2_ESM.docx]

**Leishmania infantum strain MHOM/FR/1978/LEM75 cytosolic NADP-malic enzyme (me) gene, partial cds** (DQ449701.1)

1 cgcaaccgct tcaccaataa gggcacagcc tttaccgcag cagagcggtc gcacatgaac

61 gtggaagggt tgctgccgcc ctctgtcgag accctcgatg atcaggtgga gcggtactgg

121 gatcagctga accgtttcaa cgagccgatc aaccgctatc agttgctgcg caacgtgcag

181 aacacgaacg tcaccctcta ctacgccatc ttgacgcggt acctgaagca gacactgccg

241 atcgtgtaca caccgaccgt cggcgaggcc tgccagcgct acggtgacct ctatcagaag

301 gaccacggac tgtacctcga cgtcgccatc aagggcaagg tgaggaagct gat**tcagaac**

361 **cttcgcaaga** **cga**acgtcga cgtcatcgtt atcaccgatg gctcccgcat tctcggcctg

421 ggcgacctcg gcgccaacgg catcg**gcatc** **agcatcggca** **agtg**ctccct gtacgtcgct

481 gcgggcggtg tgaagccgag ccgcgttctg ccggtcgtca tggacgttgg cacaaacaac

541 ctcgagctcc gcaacaaccc gctttatctc ggtttgcgca agccgcggtg cggcgacgcc

601 gacttttacg ctctgctgga cgagttcatg gaagctgtga aggacacctg gccctccgct

661 gtcgtgcagt tcgaggactt cagcaacaac cactgcttcg acatgctgga gcgctaccaa

721 aagaagtacc gctgcttcaa cgacgatatc cagggcaccg gcgccgtcat agctgctggt

781 ttccacacgg cggtgaagct aagcaagatc ccgatggagc agcagcgcat cgtcttcttc

841 ggcgccggct ctgccgcgac cggtgtggcg gagagcatcg ccgacctcgc cgctgaggcc

901 gggatgaaga aagaggacgt caagaagagc atcttcttcg tcgactcgat gggcatggtg

961 gccaccaacc gcggtgacaa gctggccaag cacaagctgg gatgggcccg caccgacatc

1021 cctgacgcag ttattgcaag cctgaagact ctcgaggacg ttgtgcgcta cgtgcggccg

1081 accgcgctca tcggcctcgg cgccaccgcc aacgtctttt cgcgcgagat tgtggagttt

1141 ctgcactcgt gctgccctca cccgatcata ttcccgctgt caaatccgtc cagcaaagcc

1201 gaaattgtgc cggcgaacgc atacaagtgg acgaacggcg atgccatcgt ggcctccggc

1261 agcccctttc ctgagacggt cgtcagcggc cgcacgctgc aaccatcgca aggcaacaac

1321 ctgtacatct tccccggggt gggtctcggc tgctgcattg ctcagccgcc gtacatcccg

1381 caggaggtgc tggtggcggc cgctgcctgc ctgagcacgc tggccacgcc ggacgacctc

1441 gccaagggac agctgtaccc gtctattgag gaggtgcgcc gcgtgtcgcg tgaggtggcc

1501 gtggcgtgta ttcagaagct acaggagctg gggctggcca aggcagatct gccagataac

1561 cgcccagacc tgatgaagct ggtgaagacg gctttctggg agccgcgcta cttgcccgag

1621 aactactacc tggagaagga gttg
