# Supplementary material for: High-resolution melting (HRM)-based detection of polymorphisms in the malic enzyme and glucose-6-phosphate isomerase genes for Leishmania infantum genotyping
Source: Parasit Vectors. 2023 Aug 14;16:282. doi: 10.1186/s13071-023-05878-y (PMC10426199; doi:10.1186/s13071-023-05878-y)
Supplement: Supplementary file 3 — Additional file 3: Figure S2. Sequence of L. infantum MHOM/FR/78/LEM75 (AJ620617.1) GPI gene and primers used for PCR-GPIext and PCR-GPI88. PCR-GPIext primers are underlined, PCR-GPI88 primers are in bold. The polymorphic position (1831A/G) is boxed. [file 13071_2023_5878_MOESM3_ESM.docx]

**Leishmania infantum pgi gene for glucose-6-phosphate isomerase, strain MHOM/FR/1978/LEM75**

**(AJ620617.1)**

1 tgaatccctt ttcaagatga gcgattatct ttcgaagttg aaggagcacg tgctggagag

61 caccgaagtc aatggatgca caccgagcat ggccacttcg actttcaatg ccccgtatga

121 ggttgcacgc aggaccaagg tgctgggagc cacggacagc agcctgttga gcttgcctgc

181 gtggaagcgc ttgcagtccc tgtacgagaa gcacggcaac gagtccatcc tttctcattt

241 tgagaacgat catcagcgct ttgggcggta ctcgattgag gttggcctgc acagcgagga

301 aaatttcctc ttcctcgact actccaagtc gcacatcaac gacgagatca aggatgcgtt

361 ggttgcgctg gccgaggaac gtggagtgcg ggcgttcgcc aaggctatgt ttgatgggca

421 gcgggtgaac tctactgaga accgcgccgt gttgcatgtg gcgctgcgca accgcagtaa

481 ccgcccgatc atcgttgacg ggaaggatgt gatgaccgat gtgaacaatg tgcttgccca

541 aatgaaggac ttcaccgaaa aggtccgcag cggagagtgg aagggtcaga cgggcaagag

601 catttccaac atagtcaaca tcgggattgg cggcagcgac cttggcccgg tcatggtgac

661 cgaggcactg aagccgttct ccaagcgcga catgcactgc tttttcgtgt ccaacgtcga

721 tgggacacac atggctgagg ttctgaagca ggtgaacctg gaggagacca tctttatcat

781 tgcaagcaag acgttcacta cacaagaaac gttgacgaat gccatgtctg cacgcaacgc

841 gctcatggac tacctcaaag caaacaacat ctcgacggat ggcgccgttg caaagcattt

901 tgttgcccta tcgaccaaca cggaaaaggt tcgcgagttt ggcattgata ccgtcaacat

961 gtttgtgttc tgggactggg tcggtggtcg ctactctgtg tggtccgcca tcggtctctc

1021 cgtgatgctt tcgatcggct acgacaactt tgtggagttc ctgactggcg cgcacgtgat

1081 ggataaccac tttgcgtctg caccgacgga gcagaacctg ccgatgatgc tggctttggt

1141 cggcatctgg tacaacaact ttttcggcgc ggagacagag gcggtgctgc cgtacgacca

1201 gtacctgtgg cgtctgccgg cctaccttca gcagctcgac atggagagca acggcaaggg

1261 cgtgaccaag aagtctggtg cagtggctgt gcagacgggc cccattatct tcggtgaggc

1321 cggcacaaat ggtcaacatg cattctacca gctcattcac cagggcacca agatcatccc

1381 gtgcgatttc attggctgcg tccaaacaca gaaccgtgtg ggcgaccacc accggatcct

1441 gatgagcaac tttttcgcgc agacggaggc gctcatggta ggaaagagtg cggaggaggt

1501 ccgccaggag ctggccaagt ctggtatgtc ggatgaggcc attcagagta tgattccgca

1561 caaaacgttt acgggaaacc gtcccagcaa ctcgatcctg gtgaatgctc ttactccgcg

1621 tgcgctgggt gctatcatcg ccatgtacga gcacaaggtt ctcgtccagg gcgcgatctg

1681 gggcatcaac agctatgacc agtggggtgt ggagcttggc aaggtgcttg ccaagtctat

1741 cttgccgcaa ctcaagtccg gcaacatcgt ctccgatcac gacggctct**a cgaacggcct**

1801 **gatcaacat**g ttcaacacgc gcgcacatct gtgaaaaagt ctcttgatgc tactatt**tag**

1861 **agctgcgaag** **tgcatgt**tct ctttccttcc tttgttggcg attcaacgga caggtagcga

1921 ggatcg
