# Supplementary figures and images for: High-resolution melting (HRM)-based detection of polymorphisms in the malic enzyme and glucose-6-phosphate isomerase genes for Leishmania infantum genotyping
Source: Parasit Vectors. 2023 Aug 14;16:282. doi: 10.1186/s13071-023-05878-y (PMC10426199; doi:10.1186/s13071-023-05878-y)

## Slide 1
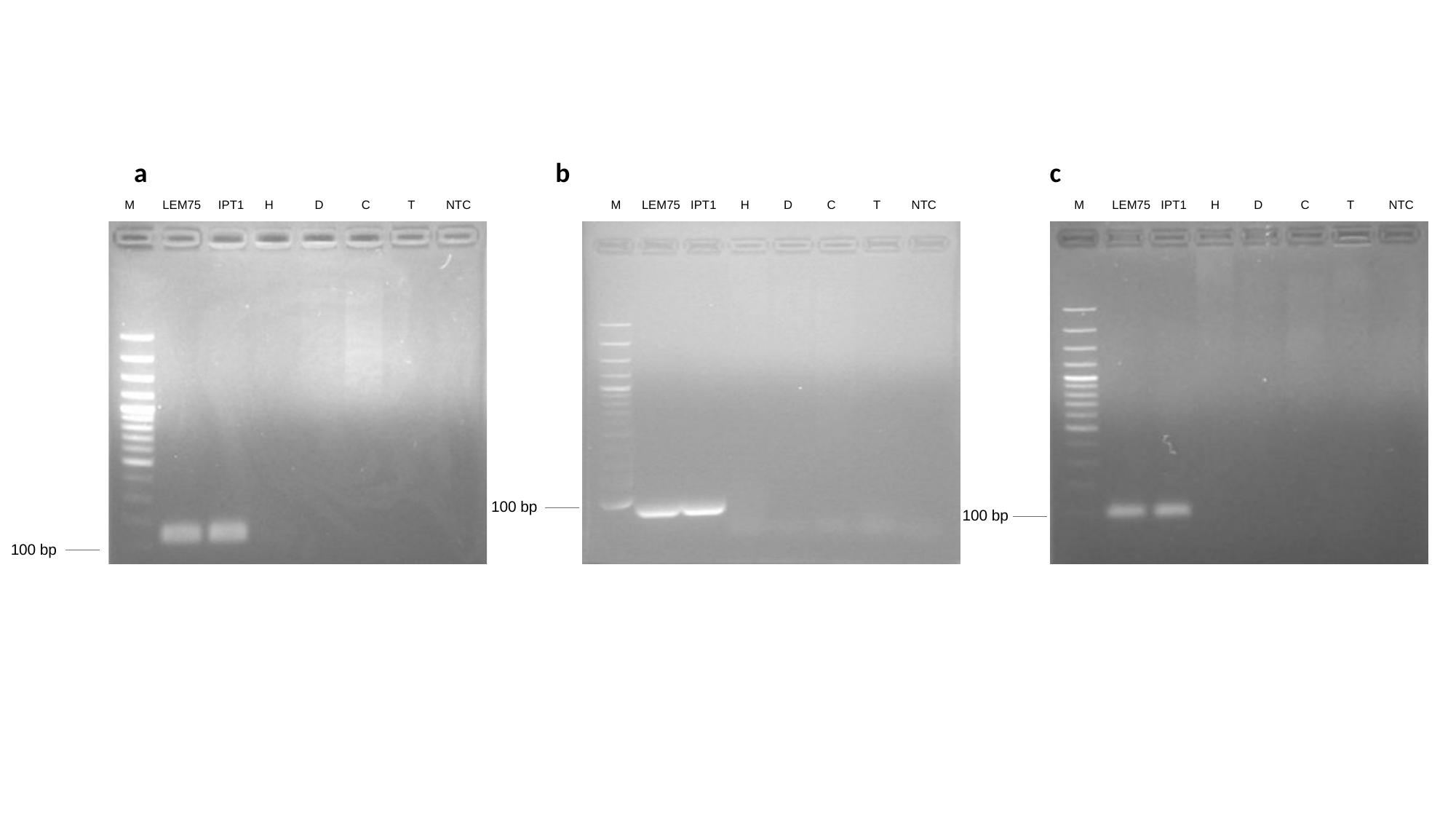

a
c
b
M LEM75 IPT1 H D C T NTC
100 bp
100 bp
M LEM75 IPT1 H D C T NTC
M LEM75 IPT1 H D C T NTC
100 bp
100 bp

Supplement: Supplementary file 4 — Additional file 4: Figure S3. Specificity evaluation of primers GPIext-F/GPIext-R (a), MEint-F/ME65-R (b) and GPI88-F/GPI88-R (c). M, marker 100-bp DNA ladder; LEM75, MHOM/FR/78/LEM75; IPT1, MHOM/TN/80/IPT1; H, human DNA; D, dog DNA; C, cat DNA; T, Trypanosoma cruzi DNA; NTC, no template control. [file 13071_2023_5878_MOESM4_ESM.pptx]

## Slide 1
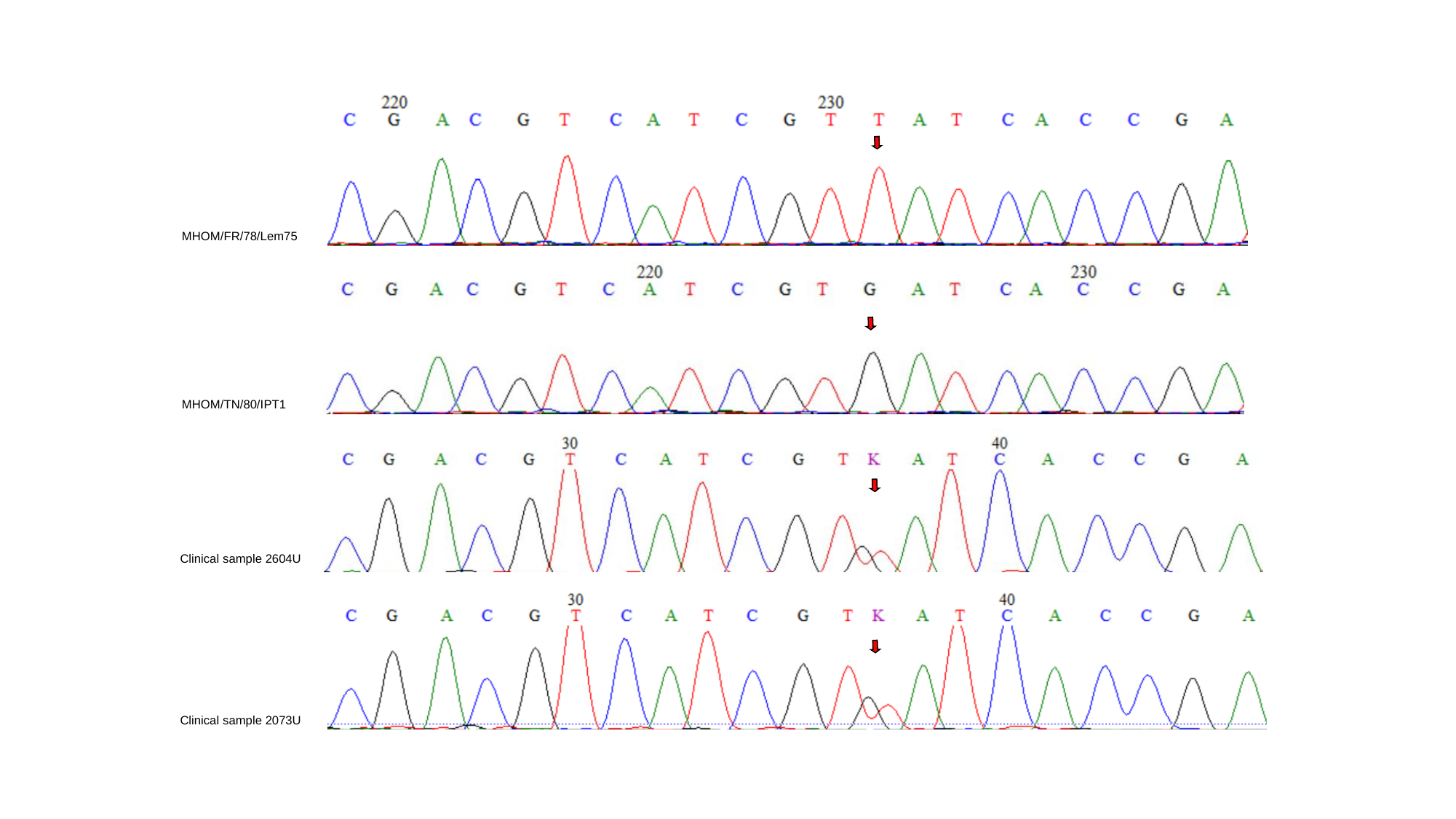

MHOM/FR/78/Lem75
MHOM/TN/80/IPT1
Clinical sample 2604U
Clinical sample 2073U

Supplement: Supplementary file 5 — Additional file 5: Figure S4. Electropherograms of clinical samples 2073U and 2604U obtained with the qPCR-MEint compared with reference strains. The arrows evidence the diagnostic SNP in position 390. [file 13071_2023_5878_MOESM5_ESM.pptx]
