# Supplementary material for: High-resolution melting (HRM)-based detection of polymorphisms in the malic enzyme and glucose-6-phosphate isomerase genes for Leishmania infantum genotyping
Source: Parasit Vectors. 2023 Aug 14;16:282. doi: 10.1186/s13071-023-05878-y (PMC10426199; doi:10.1186/s13071-023-05878-y)
Supplement: Supplementary file 6 — Additional file 6: Figure S5. Electropherograms of selected amplicons obtained with the qPCR-GPIext. The arrows indicate the position 1831 where a diagnostic SNP was found. In particular, the heterozygosis of MHOM/IT/93/ISS822 strain and clinical isolate V2921 is evident. Moreover, the electropherograms demonstrate different results in left and right conjunctival swabs of canine clinical sample Els-mai. Els-maia, Left conjunctival swab; Els-maib, right conjunctival swab. [file 13071_2023_5878_MOESM6_ESM.pptx]

## Slide 1
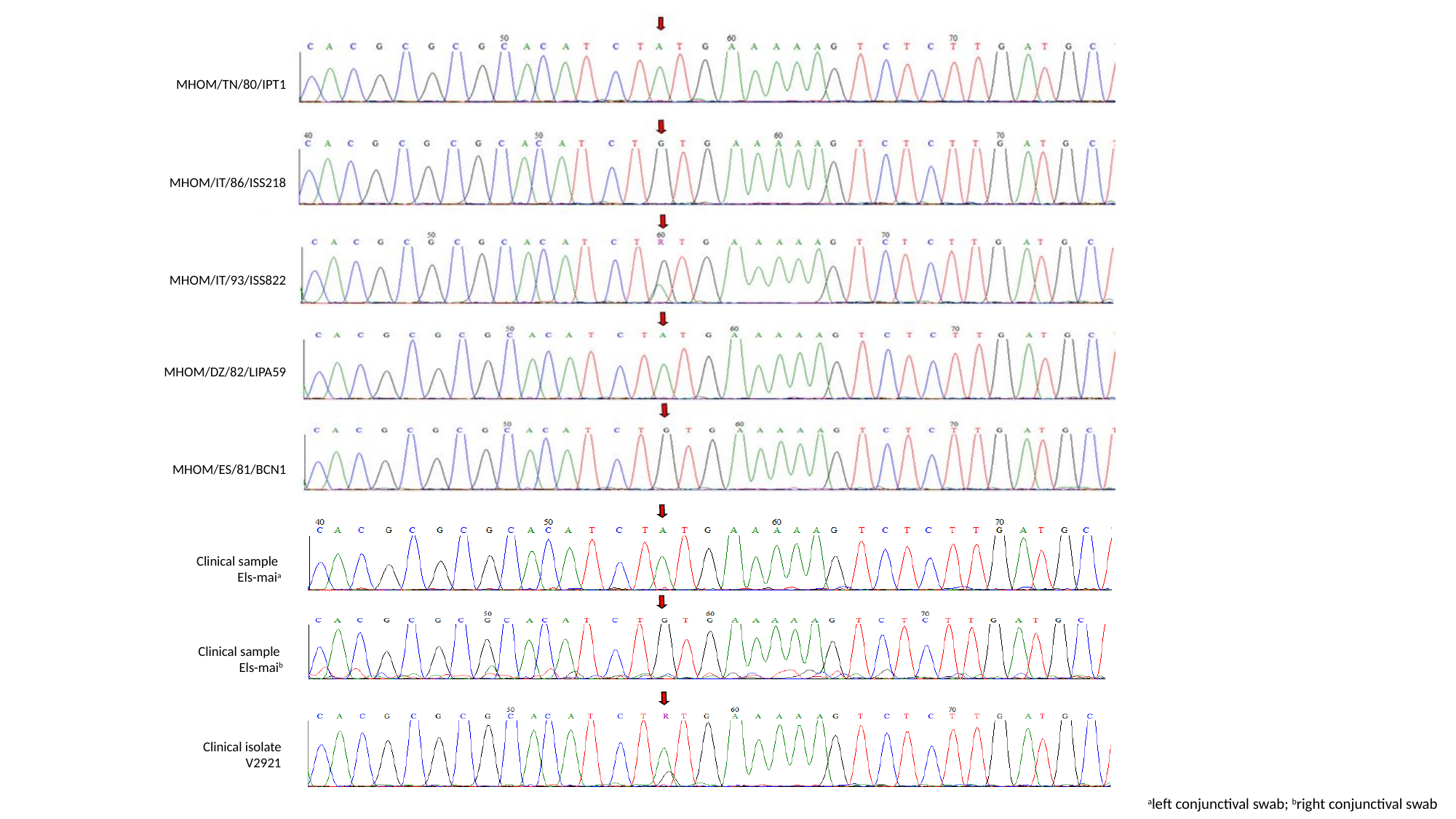

Clinical sample
Els-maia
Clinical sample
Els-maib
Clinical isolate V2921
MHOM/TN/80/IPT1
MHOM/IT/86/ISS218
MHOM/IT/93/ISS822
MHOM/DZ/82/LIPA59
MHOM/ES/81/BCN1
aleft conjunctival swab; bright conjunctival swab
